# Design and synthesis of novel nitrothiazolacetamide conjugated to different thioquinazolinone derivatives as anti-urease agents

**DOI:** 10.1038/s41598-022-05736-4

**Published:** 2022-02-07

**Authors:** Marzieh Sohrabi, Mohammad Nazari Montazer, Sara Moghadam Farid, Nader Tanideh, Mehdi Dianatpour, Ali Moazzam, Kamiar Zomorodian, Somayeh Yazdanpanah, Mehdi Asadi, Samanesadat Hosseini, Mahmood Biglar, Bagher Larijani, Massoud Amanlou, Maliheh Barazandeh Tehrani, Aida Iraji, Mohammad Mahdavi

**Affiliations:** 1 Endocrinology and Metabolism Research Center, Endocrinology and Metabolism Clinical Sciences Institute, Tehran University of Medical Sciences, Tehran, Iran; 2 Department of Medicinal Chemistry, Faculty of Pharmacy and Pharmaceutical Sciences Research Center, Tehran University of Medical Sciences, Tehran, Iran; 3 Stem Cells Technology Research Center, Shiraz University of Medical Sciences, Shiraz, Iran; 4 Department of Medical Mycology and Parasitology, School of Medicine, Shiraz University of Medical Sciences, Shiraz, Iran; 5 Department of Pharmaceutical Chemistry, School of Pharmacy, Shahid Beheshti University of Medical Sciences, Tehran, Iran; 6 Department of Medicinal Chemistry, Faculty of Pharmacy, Tehran University of Medical Sciences, Tehran, Iran; 7 Central Research Laboratory, Shiraz University of Medical Sciences, Shiraz, Iran; 8 Liosa Pharmed Parseh Company, Shiraz, Iran

**Keywords:** Chemical biology, Chemistry

## Abstract

The present article describes the design, synthesis, in vitro urease inhibition, and in silico molecular docking studies of a novel series of nitrothiazolacetamide conjugated to different thioquinazolinones. Fourteen nitrothiazolacetamide bearing thioquinazolinones derivatives (**8a-n**) were synthesized through the reaction of isatoic anhydride with different amine, followed by reaction with carbon disulfide and KOH in ethanol. The intermediates were then converted into final products by treating them with 2-chloro-N-(5-nitrothiazol-2-yl)acetamide in DMF. All derivatives were then characterized through different spectroscopic techniques (^1^H, ^13^C-NMR, MS, and FTIR). In vitro screening of these molecules against urease demonstrated the potent urease inhibitory potential of derivatives with IC_50_ values ranging between 2.22 ± 0.09 and 8.43 ± 0.61 μM when compared with the standard thiourea (IC_50_ = 22.50 ± 0.44 μM). Compound **8h** as the most potent derivative exhibited an uncompetitive inhibition pattern against urease in the kinetic study. The high anti-ureolytic activity of **8h** was confirmed against two urease-positive microorganisms. According to molecular docking study, **8h** exhibited several hydrophobic interactions with Lys10, Leu11, Met44, Ala47, Ala85, Phe87, and Pro88 residues plus two hydrogen bound interactions with Thr86. According to the in silico assessment, the ADME-Toxicity and drug-likeness profile of synthesized compounds were in the acceptable range.

## Introduction

The urease (urea amidohydrolase EC 3.5.1.5) is a Ni-containing enzyme that catalyzes the hydrolysis of urea (CH_4_N_2_) into ammonia (NH_3_) and carbon dioxide (CO_2_). The excess release of ammonia significantly increases the pH level and contributes to pathogen-host interactions^1,2^. In more detail, increasing the pH by the accumulation of NH_3_ makes the conditions more favorable for bacterial growth and development as well as increases infections of the gastrointestinal tracts and urinary system. Additionally, severe complications may occur, such as peptic ulcers, stomach cancer, hepatic coma, hepatic encephalopathy, urinary stones, catheters blocking urolithiasis, urinary catheter encrustation, and pyelonephritis^3,4^.

The possible reaction mechanism for urease activities at neutral pH involves the coordination of H_2_O–Ni plus hydroxyl groups to other Ni. Next, the substrate (urea) is activated toward nucleophilic attack by O-coordination of Ni^2+^ ions, and a nickel-coordinated hydroxide ion attacks the carbonyl carbon of the coordinated substrate to form a tetrahedral intermediate. The breakdown of the tetrahedral intermediate happened to form a coordinated carbamate or carboxylate ion. Finally, the replacement of the coordinated carbamate ion or carboxylate ion by water leads to the regeneration of the enzyme^5–7^.

One of the most frequently studied bacteria related to urease is *Helicobacter pylori* (*H. pylori),* which colonizes more than half of the human population. Urease of *H. pylori* as a virulence factor neutralizes the acidic pH of the stomach, leading to alteration of the properties of the gastric mucous layer^8^ as well as providing ammonia for bacterial protein synthesis. Urease can induce destructive effects on host tissues directly by the produced ammonia and indirectly through stimulation of inflammation and immune response, including recruitment of leukocytes and triggering of the oxidative burst in neutrophils^9,10^. Specifically, *H. pylori* infection can induce and modulate the synthesis of angiogenic and invasive factors in gastric cancer cells^11^.

Urease inhibition can be a major strategy to target diseases associated with urease. To design effective urease inhibitors, the structure and active site of the aforementioned enzyme should be discussed. The urease consists of four domains: the N-terminal αβ domain (1–134 residue), the second αβ domain (135–285), β domain (286–401 and 702–761 located in the middle of 3D structure), and the C-terminal (αβ)8 TIM barrel domain (402–701 plus 762–840). (αβ)8 TIM barrel domain contains a flap region and an active site in which Ni1 and Ni2 are separated by a distance of less than 4 Å. Residues His519, His545, and Lys490 are connected to Ni1, while the residues His407, His409, Asp633, and Lys490 are linked to Ni2^12^. The flap pocket modulates the entrance of urea into the active site of the enzyme^13^. The structures of potent inhibitors displayed the critical role of interaction with Ni (I) and Ni (II) as well as the residues of the binding site.

Several urease inhibitors with various structures have been introduced, including dihydropyrimidine thiosemicarbazones^14^, sulphamethazine, sulphamethoxazole^15^, hydroxamic acids^16^, thiobarbiturate^17,18^, bis-indole^19^, benzofuran^20^, sulfonated-coumarin^21^ benzimidazole^22^, thiazolidinone^23^, thiosemicarbazide^24^, as well as quinazoline-4(3H)-one^25^.

Nitrogen-containing heterocycles have attracted considerable attention due to their wide occurrence and pharmacological importance. Among these heterocycles, quinazoline and quinazolinone-based derivatives, constitute an imperative class of compounds with various methodologies for their synthesis, such as aza-reaction, metal-mediated (Pd, Zn, Cu) reaction, microwave-assisted reaction, ultrasound-promoted reaction, and phase-transfer catalysis. All these strategies provide rapid access to novel quinazoline and quinazolinone derivatives, affording the possibility of increasing structural diversity to the design and synthesis of novel agents with diverse therapeutic and pharmacological properties^26,27,27,28,28,29^. Considerable evidence has been found on the importance of quinazolinone derivatives in pharmaceutical chemistry as an important nucleus in the class of anticancer^29^, anti-inflammatory^30^, anticonvulsant, antihypertensive^31^, antidiabetic^32^, and antimicrobial agents^33^. Besides quinazolinone derivatives also exhibited significant urease inhibitory potencies^34,35^, but further development is required to find a lead with the quinazolinone-based structure for future advanced research. Aminonitrothiazole scaffold is also known as an important class of antibacterial agents^36–38^. By considering the rapid increase of resistance to existing drugs, a vital need for new candidates possessing urease inhibitory activity as one of the key virulence factors in the human pathogen is highly needed. Keeping this in view, in the present study, structural modifications to the previously reported quinazolinone as the elegant skeleton against urease via coupling to aminonitrothiazole were considered to evaluate the potency of newly synthesized derivatives. This manuscript describes the synthesis of nitrothiazolacetamide conjugated to thioquinazolinone **8a-n** and the evaluation of their urease inhibition activities. Moreover, a structure–activity relationship (SAR) was established, followed by a mechanism of action and molecular modeling evaluations of potential inhibitors.

## Results and discussion

### Designing consideration

**Figure 1:**
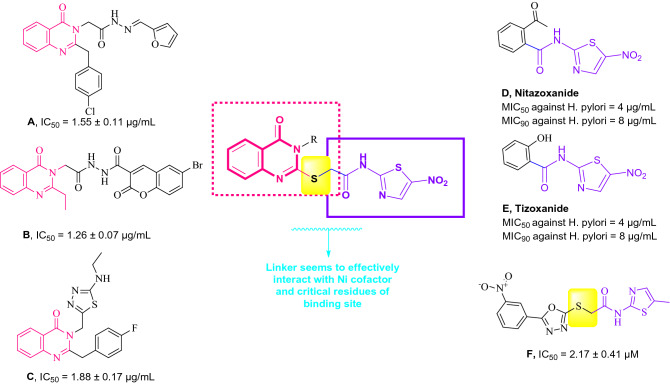
Chemical structures of some biologically active agents and commercial medicine against urease.

For the past few years, there are limited reports of quinazolines as urease inhibitors. New series of 2,3-disubstituted quinazolin-4(3H)-ones (Fig. 1A) were synthesized and exhibited potent urease inhibitory activity in the range of 1.55–2.65 μg/mL. The structure–activity relationship (SAR) indicated that halogen atoms on phenyl ring improved urease inhibition^34^. More recently, Mustafa and co-workers identified another set of quinazolinone-coumarin derivatives and the most potent compound (Fig. 1B) exhibited the IC_50_ values of 1.26 ± 0.07 μg/mL. In vitro results showed that the heterocyclic group substituted on N-3 position of quinazolinone ring plays an important role in the inhibitory activity^39^. This research group also developed and synthesized another series of quinazolin-4(3*H*)-ones (Fig. 1C). Most of the compounds showed excellent activity with IC_50_ values ranging between 1.88 ± 0.17 and 6.42 ± 0.23 μg/mL, compared to that of thiourea with an IC_50_ value of 15.06 μg/mL. Molecular docking interactions of compound **C** as the most potent derivative of this set showed key interactions with Arg439, Met637, Gln635 residues^35^.

Nitazoxanide (Fig. 1D) and nitazoxanide (Fig. 1E) are known as approved antiparasitic medications with aminonitrothiazole structure. These compounds were shown to have antibacterial activities against both metronidazole-resistant strains and sensitive clinical isolates of *H.* *pylori* pathogens. It is noteworthy that strains resistant to metronidazole were susceptible to these drugs^40^. Recently, thiazolbenzamide (Fig. 1F) was reported as a potential urease inhibitor. It was inferred that molecules with thio-substituted groups generally improved the urease inhibition of the target enzyme^41^.

Also, it would be interesting to note that the most effective inhibitors contain functional groups with electronegative atoms such as oxygen, nitrogen, or/and sulfur to form complexes with Ni ions of the enzyme as well as His residues in the active site. Stronger interaction of inhibitors with enzyme active site and higher inhibitory efficiency was observed in sulfur-containing inhibitors compared to the rest of heteroatoms ^42,43^.

By considering the structure of the previously reported active agents discussed herein.

- Quinazolinone was utilized as an elegant skeleton to design urease inhibitors. Substitution at the R position of quinazolinone was performed to evaluate the type of substitution against urease.
- To improve urease inhibitory potency, the nitrothiazole pendant with ensured anti-urease properties was incorporated into the quinazolinone ring. Nitrothiazole can improve hydrogen bonding capability within the enzyme cavity.
- Thioacetamide is an ideal candidate to link the quinazolinone and nitrothiazol moiety with the substrate-like structure. It was assumed that sulfur atoms provide better and sometimes selective interactions with critical Ni (I) and Ni (II) coordinated with His519, His545, Lys490, His407, His409, Asp633, and Lys490^45^.

In continuation of our previous effort on designing urease inhibitors^46–48^, this work was aimed to report the synthesis of nitrothiazolacetamide conjugated to different thioquinazolinones. The urease inhibitory potential of all derivatives, as well as SAR and molecular docking studies, were also performed.

### Chemistry

**Figure 2:**
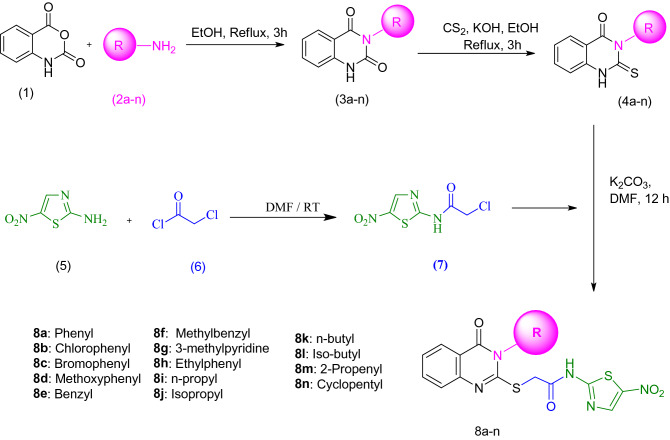
The synthetic path of the target compounds **8a-n**.

The synthetic pathway to the target compounds (**8a-n**) is outlined in Fig. 2. Intermediates **3a-n** were synthesized by the method reported in our previous study^44^. Briefly, isatoic anhydride (**1**) was reacted with different amine (**2a-n**) in ethanol under reflux conditions for 3 h to obtain compound **3a-n**. Carbon disulfide and KOH were added to this solution and the reaction was further refluxed for an extra 3 h. The targeted compounds (**4a-n**) were obtained after cooling and recrystallizing in ethanol. Compound **7** was prepared by a simple reaction of nitrothiazolamine (**5**) with 2-chloroacetyl chloride (**6**) in DMF at room temperature. The crude product was purified by recrystallization in ethanol. Compounds **8a-n** were synthesized by the nucleophilic addition of thio-derivatives (**4a-n**) to intermediate **7** in DMF using K_2_CO_3_ as a catalyst at 50 °C. The structures of purified products were confirmed by IR, ^1^H NMR, ^13^C NMR, elemental analysis, and mass spectroscopy.

### Evaluation of urease inhibitory activity and structure–activity relationship

In vitro anti-urease activity of synthesized compounds, **8a-n** were performed based on the calorimetric method against urease compared with thiourea as the reference inhibitor. The results of the urease inhibitory assay were shown in Table 1 in the terms of IC_50_. In this series, all compounds had significant inhibition against urease with IC_50_ values ranging from 2.22 to 8.43 µM compared with thiourea as a positive control with an IC_50_ value of 22.50 µM.

**Table 1:**
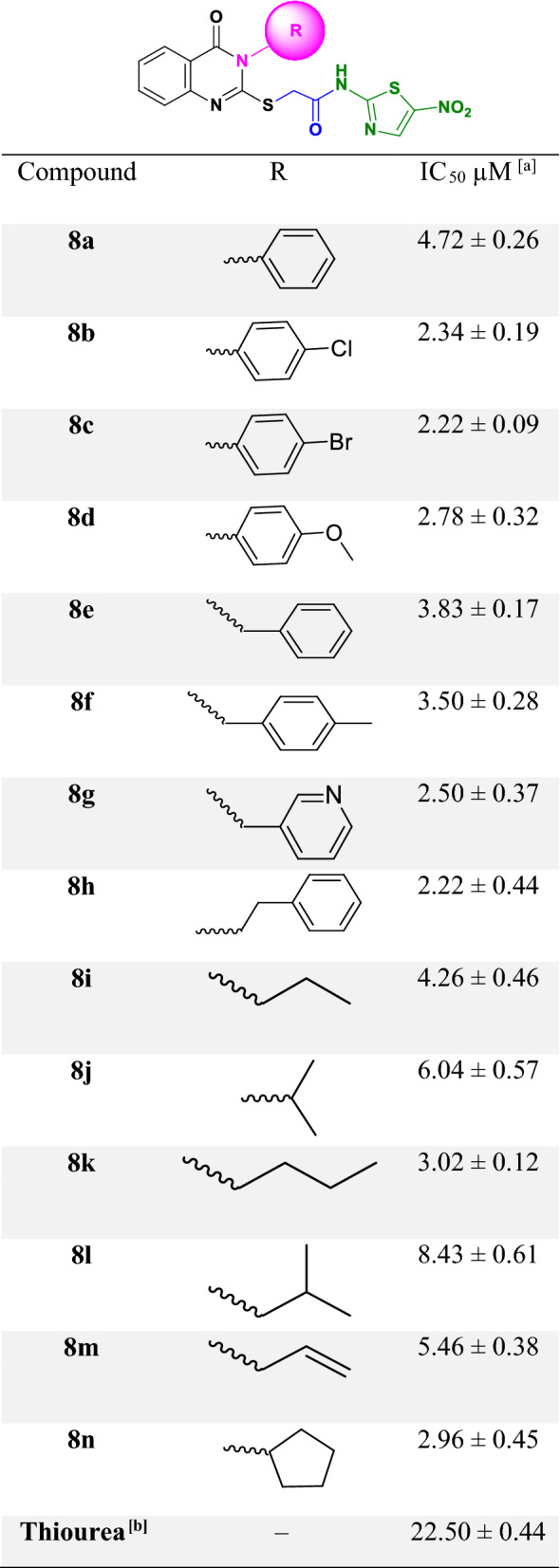
Urease inhibitory activity of the nitrothiazole thioacetamide containing different quinazolinone moieties.

^a^IC_50_ values are expressed as mean ± standard error of three independent experiments.

^b^Standard inhibitor of urease.

Based on the obtained biological results related to **8a-d**, compound **8a** as the unsubstituted phenyl pendant displayed an IC_50_ value of 4.72 µM with around fivefold improvement in the potency compared to thiourea as a standard inhibitor. Any substitution in this group including electron-withdrawing such as chlorine (**8b**) or bromine (**8c**) or even electron-donating group (**8d**, methoxy) improved urease inhibition and there is snot significant differences in these substituted derivatives.

The evaluations on **8e-g** as the methyl-substituted group demonstrated that **8 g** (R = methylpyridine) with an IC_50_ of 2.50 μM was categorized as the top potent urease inhibitor in this group followed by **8f** (R = methyl benzyl) and **8e** (R = benzyl). It seems that the presence of heteroatom in the aromatic ring could amend the interactions within the binding site of urease.

Assessments of **8a**, **8e,** and **8 h** analogs showed the importance of the length of the alkyl chain between quinazolinone and aryl moiety. Compound **8h** bearing ethyl linker demonstrated an IC_50_ of 2.22 μM against urease, while **8e** (IC_50_ = 3.83 μM) possessing methyl linker was less potent compared with **8h** followed by **8a** with IC_50_ = 4.72 μM. It seems that the elongation of the alkyl linker between the quinazolinone and aryl pendant improved urease inhibitory activity.

In the case of compounds containing aliphatic chain substitution (**8i-m**), it can be seen that in most cases such structural modification reduced the inhibitory potency of compounds (IC_50_ ranging from 3.02 to 8.43 μM) compared to aromatic substituted derivatives (IC_50_ ranging from 2.22 to 3.85 μM). In this group, the most potent urease inhibitor was **8 k** (R = n-butyl) with an IC_50_ value of 3.02 µM followed by **8i** (R = n-propyl; IC_50_ = 4.26 μM), **8j** (R = iso-propyl; IC_50_ = 6.04 μM) and **8l** (R = iso-butyl; IC_50_ = 8.43 μM). As can be seen, the longer aliphatic chain demonstrated better inhibitory activity compared to shorter or branch one. Interestingly, compound **8n** bearing cyclopentyl group as an aliphatic-ring substitute showed better activity (IC_50_ = 2.96 μM) compared to the rest of the aliphatic-chain group. As can be seen in this set of compounds, it seems that aliphatic-ring followed by aliphatic linear chains are more potent than aliphatic branched-chain counterparts.

### Kinetic study of the most potent compound 8h

**Figure 3:**
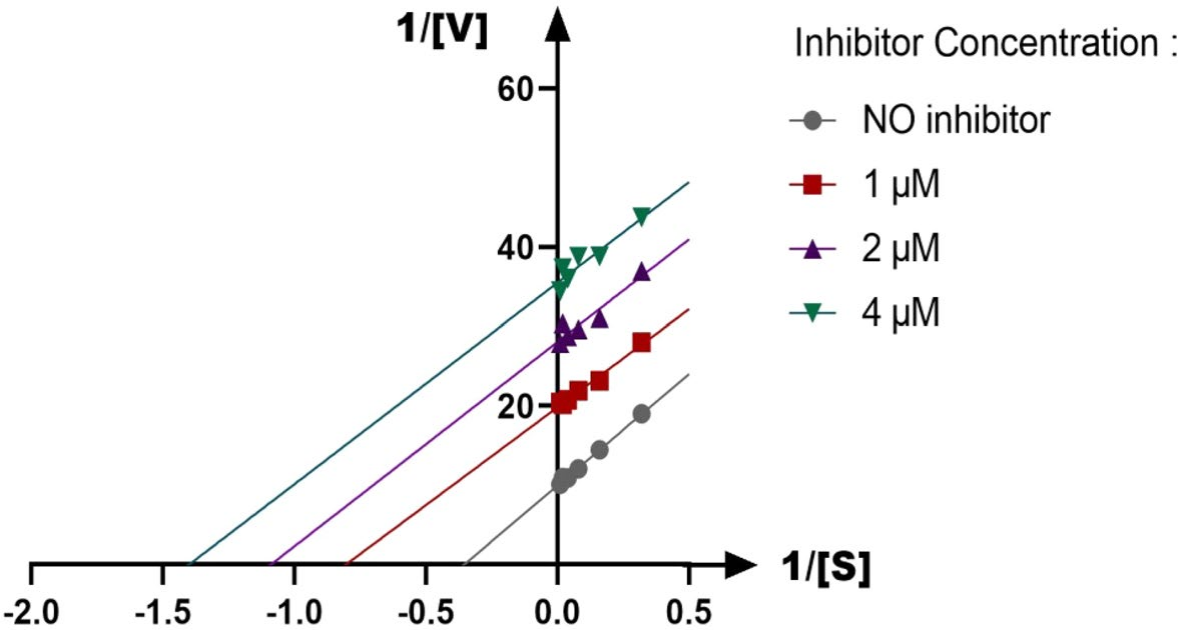
The Lineweaver–Burk plot of compound **8h** at different concentrations against urease of three independent experiments.

**Figure 4:**
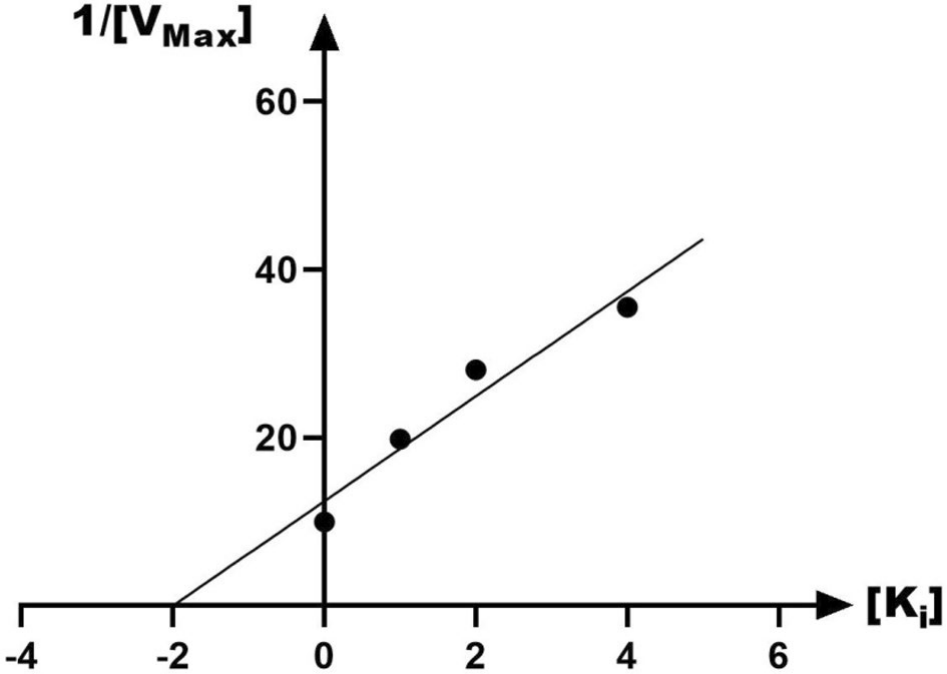
Double reciprocal Lineweaver–Burk plot of **8h** against urease of three independent experiments.

The mechanism of urease inhibition was investigated by enzyme kinetics, following the similar procedure of the urease inhibition assay. Lineweaver–Burk graphics were used to estimate the type of inhibition. Graphical analysis of the reciprocal Lineweaver–Burk plot (Fig. 3) related to compound **8h** showed that K_m_ and V_max_ decreased with an increase in inhibitor concentration confirming an uncompetitive inhibition pattern against urease. Furthermore, the plot of the K_m_ versus different concentrations of **8h** gave an estimate of the inhibition constant, K_i_ of 1.994 µM which is in accordance with the IC_50_ value of **8h** (Fig. 4).

### Molecular docking simulation

**Figure 5:**
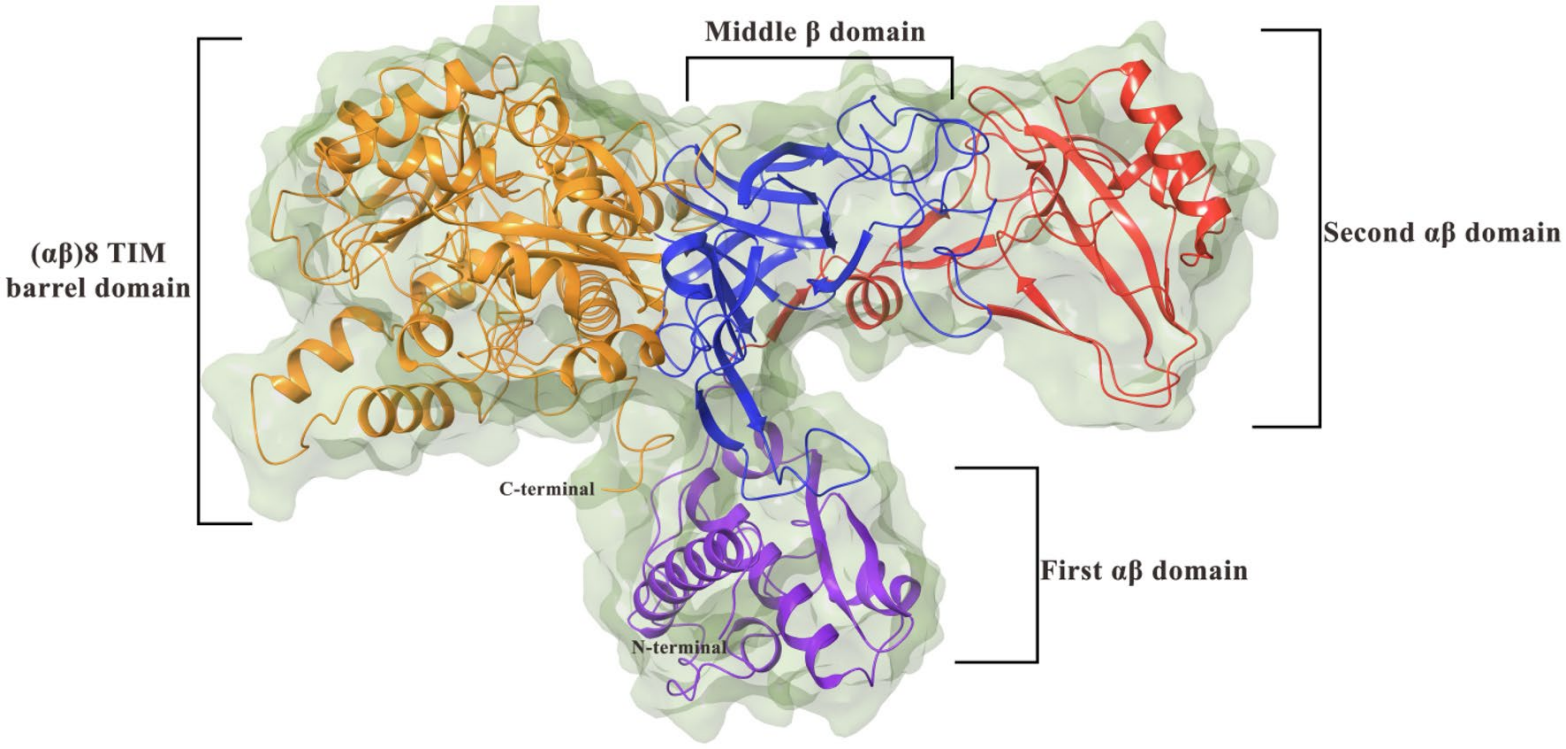
Schematic view of jack bean urease domains.

Jack bean urease (*JBU*) is a T-shaped metalo-hydrolase enzyme that acts by converting urea into ammoniac within its active site. *JBU* monomer third structure consists of four main domains (Fig. 5). From the N-terminal of the enzyme sequence, starts by first αβ domain located in the hammer handle. The second αβ domain is located in the hammerhead which is connected through a middle β domain to the other head of the hammer which is (αβ)_8_ TIM barrel domain holding the active site of the enzyme^12^.

The enzyme kinetic study showed that the compound **8h** acts as an uncompetitive inhibitor of the *JBU* enzyme in this type of inhibition the inhibitor interacted with the enzyme–substrate ([ES]) complex to form a final enzyme–substrate-inhibitor ([ESI]) complex; hence, the molecular docking study was performed on the [ES] complex. To make the [ES] complex the urea docked into the active site of the *JBU* enzyme (PDB ID: 4H9M).

**Figure 6:**
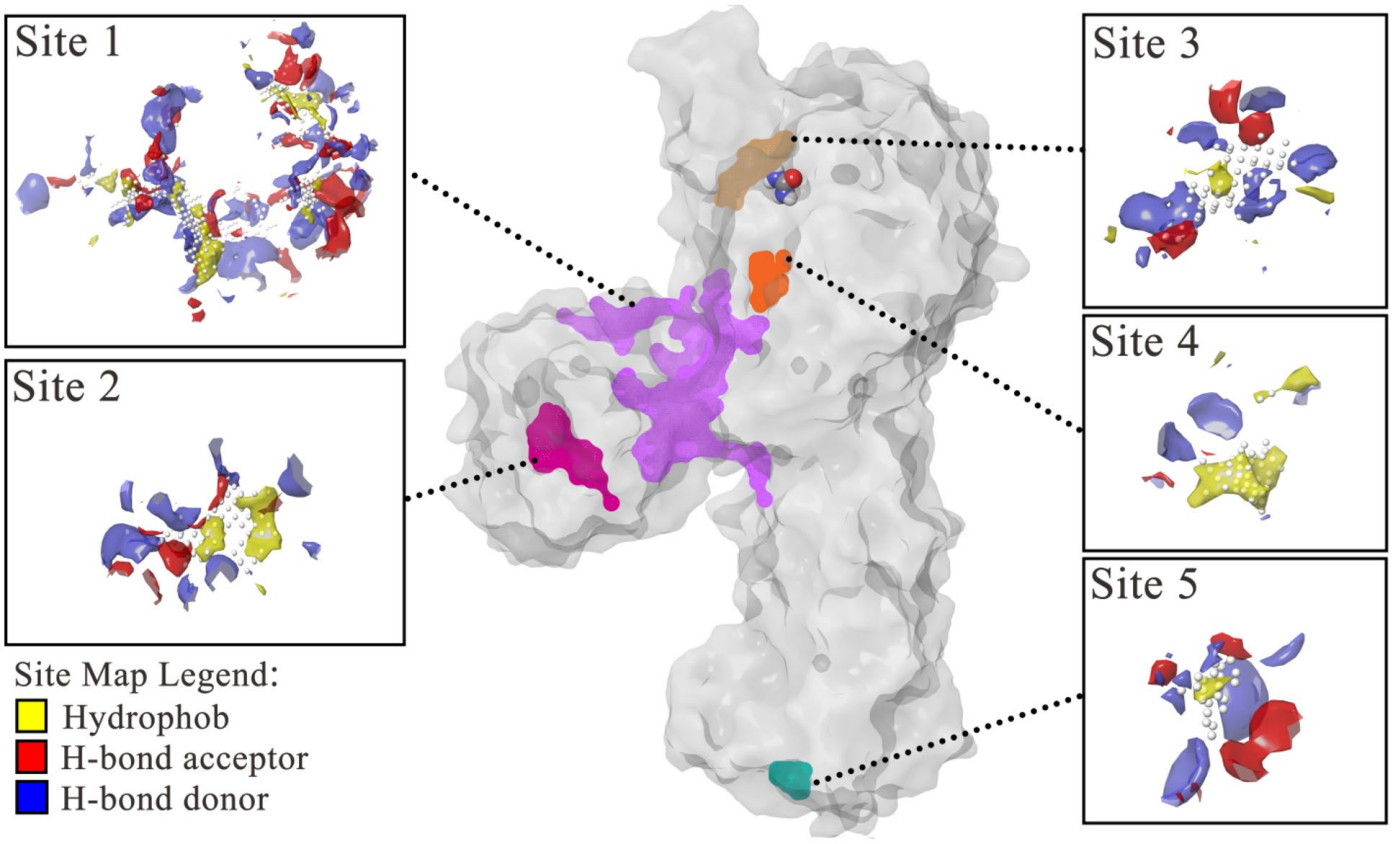
Potential binding sites of jack bean urease detected by sitemap. In each site’s magnified image the hydrophob (yellow), H-bond acceptor (red), and H-bond donor (blue) parts have been indicated.

In order to find the possible allosteric sites, the protein-substrate complex was treated using mastreo sitemap tool to identify the suitable sites for occupancy of hydrophobic, H-bond donor, and H-bond acceptor ligand groups. Eventually, five possible binding sites were detected which can be suitable as a drug-like binding site. As shown in Fig. 6, five binding sites were detected on the surface of the [ES] complex. Site 1 (purple) and site 2 (magenta) located in the first αβ domain which was showed the suitable size and potential interaction sites, calculated to have the best site scores (1.011 and 0.932 respectively). Site 3 (brown) nearby the canonical active site also had a plausible site score of 0.917. Site 4 (orange) and site 5 (cyan) were the smallest sites and had a few potential H-bond interactions, their site scores were calculated to be 0.724 and 0.510, respectively.

**Figure 7:**
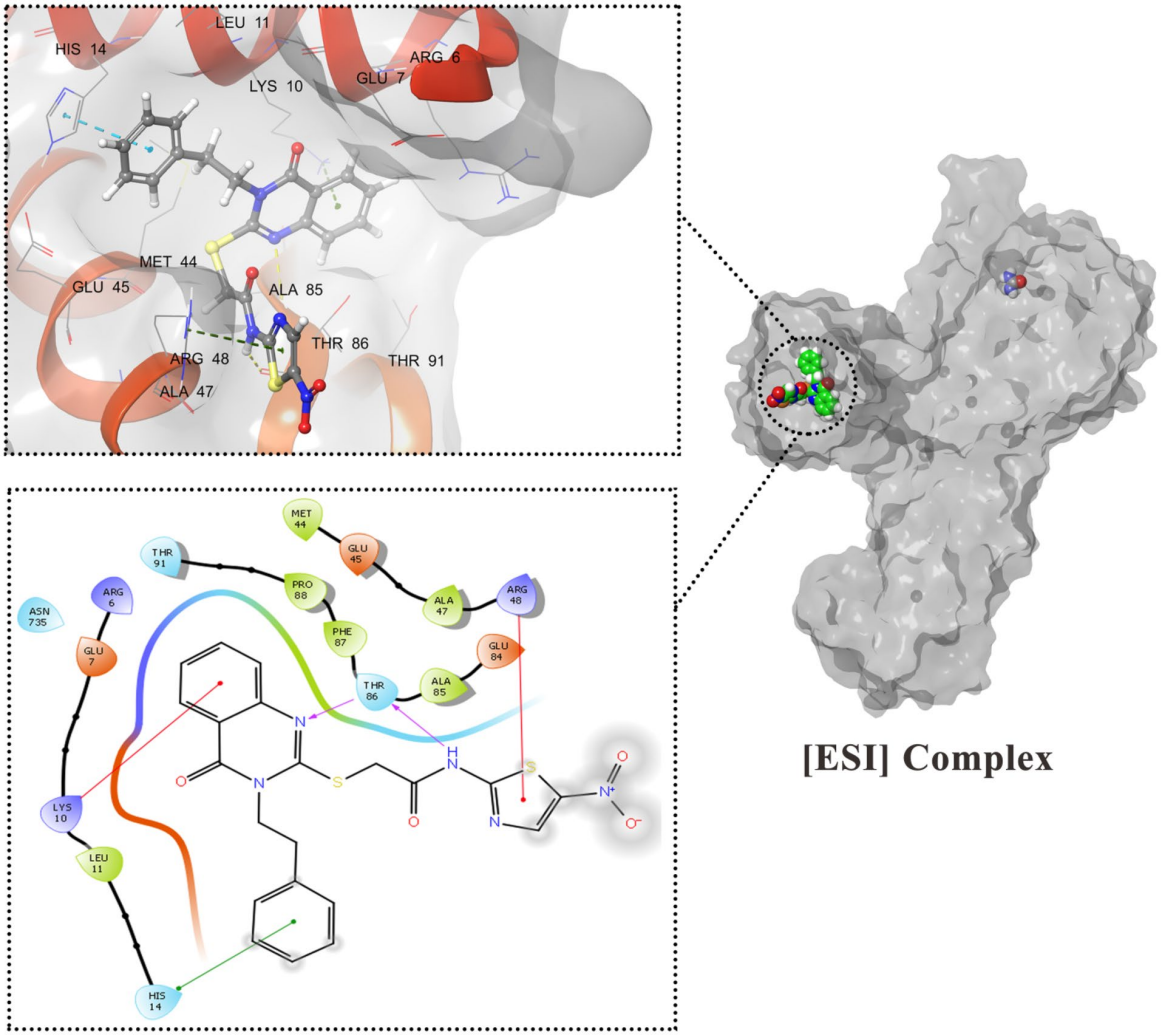
3D and 2D interactions of compound **8h** in the [ESI] complex.

Compound **8h** as the most potent structure in the series, was docked on all of the potential binding sites of the [ES] complex to form the enzyme–substrate-inhibitor [ESI] complex. Considering the glide score (− 6.78 kcal/mol) and interactions, site 2 appeared to have the maximum affinity in comparison with other identified sites. As it is shown in Fig. 7, compound **8h** well occupied the site, and the following interactions were detected: Thr86 residue acting as both H-bond acceptor and H-bond donor with amide group and quinazolinone ring nitrogen. The pi-cation interaction between Lys10 and the quinazolinone aromatic system and another pi-cation interaction between Arg48 and the thiazole ring was observed. A pi-pi stacking interactions were found among His14 and ethylbenzene moiety moreover several hydrophobic interactions were found between compound **8h** and Leu11, Met44, Ala47, Ala85, Phe87, and Pro88 residues.

### Antimicrobial and anti-ureolytic activity of tested compounds

Compounds **8c**, **8g**, and **8h** were chosen for their antimicrobial activities against microorganisms including standard species of *Cryptococcus* *neoformans* (H99), and clinical isolate of *Proteus* *vulgaris*. The results showed that at concentrations ranging from 1 to 512 μg/ml, the examined compounds exhibited no antimicrobial activities against the tested pathogens (MIC > 512 (μg/ml).

Next, the anti-ureolytic activity of highly potent urease inhibitors (**8c**, **8g**, and **8h**) against the *C. neoformans* (H99) and *P.vulgaris* was visually and spectroscopically measured at 560 nm. Table 2 summarizes the findings. Compound **8h**, like our enzymatic assay results, displayed the highest anti-ureolytic activities followed by compound **8c**. Notably, **8g** exhibited selective urease activity against *C. neoformans* but not against *P.* *vulgaris* at the tested range.

**Table 2:** Anti-ureolytic effects of selected compounds against *C. neoformans* and *P.vulgaris.*

Ureolytic organism	IC_50_ (µg/ml) of *C.* *neoformans*	IC_50_ (µg/ml) of *P.* *vulgaris*
**Compound**		
**8c**	173.8 ± 4.9	383.3 ± 5.1
**8g**	241. 8 ± 7.1	> 512
**8h**	129.4 ± 5.3	172.4 ± 8.7

According to the findings, none of the selected derivatives had anti-microbial effects on the tested microorganisms; however, the high activity of tested compounds against ureolytic microorganisms strengthens our hypothesis that the designed pharmacophore can be an ideal candidate for targeting ureolytic microorganisms through urease enzyme inhibition.

### ADME-toxicity profiles and physicochemical properties

The pkCSM server^45^ was used to predict the ADME-Toxicity properties of synthesized compounds. As shown in Table 3. All derivatives showed good human intestinal absorption, low clearance values, and limited toxicity.

**Table 3:** ADMET prediction of the synthesized derivatives as urease inhibitors.

	Absorption	Distribution	Metabolism							Excretion	Toxicity
	Human intestinal absorption (% absorbed)	VDss (logL/Kg)	2D6	3A4	1A2	2C19	2C9	2D6	3A4	Total clearance (log mL/min/kg)	Oral rate acute toxicity (mol/kg)
			Substrate		Inhibitor						
**8a**	93.821	− 0.71	No	Yes	Yes	Yes	Yes	No	Yes	0.091	2.642
**8b**	94.839	− 0.607	No	Yes	Yes	Yes	Yes	No	Yes	− 0.042	2.647
**8c**	94.575	− 0.595	No	Yes	Yes	Yes	Yes	No	Yes	− 0.063	2.649
**8d**	90.757	− 0.648	No	Yes	Yes	Yes	Yes	No	Yes	0.125	2.648
**8e**	92.759	− 0.618	No	Yes	Yes	Yes	Yes	No	Yes	0.136	2.749
**8f**	93.153	− 0.455	No	Yes	No	Yes	Yes	No	Yes	0.084	2.746
**8g**	85.389	− 0.036	No	Yes	No	Yes	No	No	Yes	0.057	2.668
**8h**	81.622	− 0.492	No	Yes	No	No	Yes	No	Yes	0.146	2.614
**8i**	87.271	− 0.249	No	Yes	No	Yes	No	No	Yes	0.097	2.565
**8j**	88.629	− 0.423	No	Yes	Yes	Yes	Yes	No	Yes	0.015	2.383
**8k**	88.208	− 0.159	No	Yes	No	Yes	Yes	No	No	0.13	2.586
**8l**	88.629	− 0.423	No	Yes	Yes	Yes	Yes	No	Yes	0.015	2.383
**8m**	86.418	− 0.259	No	Yes	No	Yes	No	No	Yes	0.149	2.548
**8n**	94.008	− 0.009	No	Yes	No	Yes	Yes	No	No	0.045	2.656

According to the physicochemical properties predicted from the SwissADME website^46^, all compounds had appropriate molecular properties with no drug-likeness rules violations (Table 4).

**Table 4:** Drug-likeness properties of synthesized compounds.

Compound	MW	Num. rotatable bonds	Num. H-bond acceptors	Num. H-bond donors	Log *P*
**8a**	439.478	6	9	1	3.4812
**8b**	473.923	6	9	1	4.1346
**8c**	518.374	6	9	1	4.2437
**8d**	469.504	7	10	1	3.4898
**8e**	405.461	7	9	1	2.902
**8f**	467.532	7	9	1	3.8487
**8g**	454.493	7	10	1	2.9353
**8h**	469.548	8	10	3	3.4113
**8i**	405.461	7	9	1	2.902
**8j**	405.461	6	9	1	3.0729
**8k**	419.488	8	9	1	3.2921
**8l**	405.461	6	9	1	3.0729
**8m**	403.445	7	9	1	2.678
**8n**	431.499	6	9	1	3.6071

## Conclusion

In summary, fourteen new compounds with thioquinazolinone structures were designed and prepared as anti-urease agents. Among them, compound **8h** exhibited the most potent inhibitory effect against urease with an IC_50_ value of 2.22 μM with around a ten-fold increase in the potency compared to the positive control. In addition, compound **8h** possessed the uncompetitive type of inhibition in the enzymatic assay indicating that ligand bonded only to the complex formed between the enzyme and the substrate. The molecular docking study revealed that compound **8h** could fit well into the binding site of urease by pi-cation, pi–pi, and H-bond interactions. **8h** also demonstrated IC_50_ values of 129.4 ± 5.3 and 172.4 ± 8.7 µg/ml against *C. neoformans* and *P.vulgaris* on the ureolytic assay. Furthermore, in silico evaluations also found acceptable ADME-Toxicity and drug-likeness profiles.

## Material and method

### Chemistry

Compounds **3a-n** were obtained by reaction of isatoic anhydride (compound **1**, 1 mmol) with different amines (compound **2**, 1.1 mmol) as the raw materials in ethanol under reflux conditions for 3 h. To the above solution carbon disulfide and KOH were added and the reaction was further refluxed for an extra 3 h to afford compounds **4a-n.** Next, the intermediate **7** were synthesized by a simple reaction of nitrothiazolamine (**5**) with 2-chloroacetyl chloride (**6**) in DMF at room temperature. Finally, compounds **4a-n** were reacted with 2-chloro-N-(5-nitrothiazol-2-yl)acetamide in the presence of K_2_CO_3_ to provide the crude products **8a-n** which was purified by column chromatography to yield the final products.

#### *N-(5-nitrothiazol*-2-yl)-2-((4-oxo-3-phenyl-3,4-dihydroquinazolin-2-yl)thio)acetamide (8a)

Brown solid; isolated yield: 80% (351 mg), mp 230–232 °C; IR (KBr) υ: 3317, 3062, 3010, 2951, 1692, 1661, 1651, 1631, 1592, 1560, 1481, 1466, 1444, 1410, 1351, 1321, 1279, 1222, 1180, 1077, 763, 719 cm^−1^. ^1^H NMR (300 MHz, DMSO-*d*_6_) δ 13.48 (s, 0.7H, exchangeable proton), 8.66 (s, 1H), 8.08 (dd, *J* = 7.9, 1.6 Hz, 1H), 7.97 (s, 0.1H, exchangeable proton), 7.81 (td, *J* = 7.4, 1.5 Hz, 1H), 7.70–7.37 (m, 7H), 4.23 (s, 2H). ^13^C NMR (76 MHz, DMSO) δ 169.71, 163.49, 161.03, 157.10, 147.45, 143.63, 141.72, 136.21, 135.46, 130.58, 130.08, 129.90, 127.12, 126.61, 126.20, 120.02, 36.79. Anal.Calcd for C_19_H_13_N_5_O_4_S_2_: C 51.93, H 2.98, N 15.94, S 14.59; Found: C 51.72, H 3.12, N 15.70, S 14.83. MS (EI, 60 eV): m/z (%): 439 (M^+^, 24).

#### 2-((3-(4-chlorophenyl)-4-oxo-3,4-dihydroquinazolin-2-yl)thio)-*N*-(5-nitrothiazol-2-yl)acetamide (8b)

Light brown solid; isolated yield: 82% (387 mg), mp 251–253 °C; IR (KBr) υ: 3330, 3086, 3014, 2921, 1693, 1669, 1649, 1628, 1601, 1561, 1523, 1496, 1479, 1424, 1354, 1336, 1299, 1241, 1176, 1010, 825, 751, 729 cm^−1^. ^1^H NMR (300 MHz, DMSO-*d*_6_) δ 13.52 (s, 0.9H, exchangeable proton), 8.68 (s, 1H), 8.08 (dd, *J* = 8.0, 1.5 Hz, 1H), 7.97 (s, 0.1H, exchangeable proton), 7.81 (td, *J* = 8.2, 1.2 Hz), 7.71 (d, *J* = 8.7 Hz, 2H), 7.62 (d, *J* = 8.7 Hz, 2H), 7.52–7.37 (m, 2H), 4.25 (s, 2H). ^13^C NMR (76 MHz, DMSO) δ 169.30, 162.85, 160.99, 156.65, 147.39, 143.47, 142.04, 135.53, 135.35, 135.12, 131.95, 130.19, 127.12, 126.68, 126.20, 120.01, 36.53. Anal.Calcd for C_19_H_12_ClN_5_O_4_S_2_: C 48.15, H 2.55, N, 14.78, S 13.53; Found: 48.43, H 2.63, N, 14.49, S 13.65. MS (EI, 60 eV): m/z (%): 473 (M^+^, 36).

#### 2-((3-(4-bromophenyl)-4-oxo-3,4-dihydroquinazolin-2-yl)thio)-*N*-(5-nitrothiazol-2-yl)acetamide (8c)

Dark brown solid; isolated yield: 84% (433 mg), mp 263–265 °C; IR (KBr) υ: 3358, 3068, 2959, 1696, 1669, 1656, 1639, 1592, 1552, 1482, 1468, 1418, 1359, 1292, 1250, 1160, 1135, 1080, 840 cm^−1^.

^1^H NMR (400 MHz, DMSO-*d*_6_) δ 13.50 (s, 0.9H, exchangeable proton), 8.67 (s, 1H), 8.06 (dd, *J* = 8.0, 1.5 Hz, 1H), 7.87–7.74 (m, 3H), 7.53 (d, *J* = 8.6 Hz, 2H), 7.46 (t, *J* = 7.4 Hz, 1H), 7.40 (d, *J* = 8.1 Hz, 1H), 4.23 (s, 2H). ^13^C NMR (101 MHz, DMSO) δ 169.34, 162.93, 160.93, 160.21, 156.55, 147.36, 143.50, 141.95, 135.53, 133.15, 132.20, 127.11, 126.67, 126.18, 124.01, 119.97, 36.55. Anal.Calcd for C_19_H_12_BrN_5_O_4_S_2_: C 44.03, H 2.33, N 13.51, S 12.37; C 44.25, H 2.18, N 13.42, S 12.51. MS (EI, 60 eV): m/z (%):) 516 (M^+^, 30).

#### 2-((3-(4-methoxyphenyl)-4-oxo-3,4-dihydroquinazolin-2-yl)thio)-*N*-(5-nitrothiazol-2-yl)acetamide (8d)

Light brown solid; isolated yield: 78% (365 mg), mp 245–247 °C; IR (KBr) υ: 3333, 3058, 2949, 1682, 1662, 1660, 1639, 1599, 1555, 1479, 1456, 1400, 1350, 1281, 1230, 1182, 1165, 1087, 758 cm^−1^. ^1^H NMR (400 MHz, DMSO-*d*_6_) δ 13.02 (s, 1H, exchangeable proton), 8.64 (s, 1H), 8.04–7.73 (m, 1H), 7.76 (d, *J* = 15.4 Hz, 1H), 7.44 (d, *J* = 7.7 Hz, 1H), 7.33 (t, *J* = 7.5 Hz, 1H), 7.18 (d, *J* = 8.3 Hz, 2H), 7.01 (d, *J* = 8.4 Hz, 2H), 4.20 (s, 2H), 3.81 (s, 3H). ^13^C NMR (101 MHz, DMSO) δ 169.45, 162.87, 160.66, 159.18, 157.68, 147.39, 143.44, 141.95, 139.96, 135.35, 130.42, 127.09, 126.52, 126.12, 119.95, 114.54, 55.73, 36.59. Anal.Calcd for C_20_H_15_N_5_O_5_S_2_: C 51.17, H 3.22, N 14.92, S 13.66; C 51.26, H 3.18, N 15.09, S 13.42. MS (EI, 60 eV): m/z (%): 469 (M^+^, 36).

#### 2-((3-benzyl-4-oxo-3,4-dihydroquinazolin-2-yl)thio)-*N*-(5-nitrothiazol-2-yl)acetamide (8e)

Brown solid; isolated yield: 81% (366 mg), mp 241–243 °C; IR (KBr) υ: 3329, 3059, 2951, 1693, 1650, 1651, 1630, 1586, 1545, 1480, 1456, 1401, 1359, 1326, 1265, 1232, 1163, 1023, 745, 710 cm^−1^. ^1^H NMR (300 MHz, DMSO-*d*_6_) δ 13.34 (s, 0.3 H, exchangeable proton), 8.66 (s, 1H), 8.11 (dd, *J* = 8.0, 1.5 Hz, 1H), 7.97 (s, *J* = 0.3 H, exchangeable proton), 7.79 (td, *J* = 8.4, 1.6 Hz, 1H), 7.47 (td, *J* = 8.2, 1.2 Hz, 1H), 7.42–7.23 (m, 7H), 5.37 (s, 2H), 4.35 (s, 2H). ^13^C NMR (76 MHz, DMSO) δ 169.77, 163.87, 161.25, 156.75, 147.06, 143.74, 141.55, 135.98, 135.48, 129.13, 128.02, 127.37, 127.16, 126.72, 126.11, 119.16, 47.60, 36.75. Anal.Calcd for C_20_H_15_N_5_O_4_S_2_: C 52.97, H 3.33, N 15.44, S 14.14; Found: C 53.26, H 3.56, N 15.24, S 14.44. MS (EI, 60 eV): m/z (%): 453 (M^+^, 33).

#### 2-((3-(4-methylbenzyl)-4-oxo-3,4-dihydroquinazolin-2-yl)thio)-*N*-(5-nitrothiazol-2-yl)acetamide (8f.)

Brown solid; isolated yield: 83% (387 mg), mp 254–256 °C; IR (KBr) υ: 3326, 3050, 2941, 1689, 1652, 1656, 1632, 1590, 1552, 1488, 1463, 1404, 1349, 1316, 1291, 1230, 1182, 1087, 758, 729 cm^−1^. ^1^H NMR (300 MHz, DMSO-*d*_6_) δ 13.07 (s, 0.4H, Exchangeable proton), 8.65 (s, 1H), 8.10 (d, *J* = 7.9 Hz, 1H), 7.97 (s, 0.6H, Exchangeable proton), 7.77 (t, *J* = 7.7 Hz, 1H), 7.45 (t, *J* = 7.9 Hz, 1H), 7.36 (d, *J* = 8.1 Hz, 1H), 7.23–7.11 (m, 5H), 5.32 (s, 2H), 4.34 (s, 2H), 2.29 (s, 3H). ^13^C NMR (76 MHz, DMSO) δ 169.76, 163.83, 161.23, 156.74, 147.04, 143.70, 141.57, 137.26, 135.42, 132.96, 129.65, 129.21, 127.75, 127.44, 127.14, 126.67, 119.17, 47.35, 36.73, 21.13. Anal.Calcd for C_21_H_17_N_5_O_4_S_2_: C 53.95, H 3.67, N 14.98, S 13.78; Found: C 53.90, H 3.75, N 14.90, S 13.65. MS (EI, 60 eV): m/z (%): 467 (M^+^, 32).

#### *N*-(5-nitrothiazol-2-yl)-2-((4-oxo-3-(pyridin-3-ylmethyl)-3,4-dihydroquinazolin-2-yl)thio)acetamide (8 g)

Brown solid; isolated yield: 86% (390 mg), mp 269–271 °C; IR (KBr) υ: 3335, 3120, 3096, 2863, 1691, 1652, 1651, 1630, 1586, 1584, 1545, 1489, 1456, 1406, 1340, 1401, 1366, 1359, 1326, 1265, 1232, 1191, 1028, 810, 721 cm^−1^. ^1^H NMR (300 MHz, DMSO-*d*_6_) δ 8.66 (s, 1H), 8.65 (s, 1H), 8.53 (dd, *J* = 8.0, 1.6 Hz, 1H), 8.10 (dd, *J* = 8.0, 1.5 Hz, 1H), 7.97 (s, 0.1 H, exchangeable proton), 7.83–7.68 (m, 2H), 7.52–7.32 (m, 3H), 5.39 (s, 2H), 4.37 (s, 2H). ^13^C NMR (76 MHz, DMSO) δ 169.52, 163.50, 161.29, 156.32, 149.32, 149.19, 147.02, 143.62, 141.74, 135.53, 135.36, 131.87, 127.14, 126.79, 126.11, 124.22, 119.18, 45.59, 36.64. Anal.Calcd for C_19_H_14_N_6_O_4_S_2_: C 50.21, H 3.11, N 18.49, S 14.11; Found: C 49.89, H 3.46, N 18.18, S 14.41. MS (EI, 60 eV): m/z (%): 454 (M^+^, 32).

#### *N*-(5-nitrothiazol-2-yl)-2-((4-oxo-3-phenethyl-3,4-dihydroquinazolin-2-yl)thio)acetamide (8 h)

Brown solid; isolated yield: 75% (350 mg), mp 235–237 °C; IR (KBr) υ: 3320, 3071, 2922, 2893, 1690, 1655, 1652, 1622, 1585, 1559, 1482, 1453, 1414, 1357, 1326, 1281, 1220, 1180, 1126, 1079, 752, 719 cm^−1^. ^1^H NMR (300 MHz, DMSO-*d*_6_) δ 13.51 (s, 0.4H, Exchangeable proton), 8.66 (s, 1H), 8.08 (d, *J* = 7.9 Hz, 1H), 7.97 (s, 0.6H, Exchangeable proton), 7.76 (t, *J* = 7.6 Hz, 1H), 7.56–7.11 (m, 8H), 4.40 (s, 2H), 4.28 (t, *J* = 8.1 Hz, 2H), 3.05 (t, *J* = 8.0 Hz, 2H). ^13^C NMR (76 MHz, DMSO) δ 169.82, 162.78, 160.75, 156.12, 146.99, 143.74, 141.58, 138.17, 135.28, 129.14, 129.13, 127.22, 126.95, 126.60, 126.04, 119.24, 46.18, 36.58, 33.91. Anal.Calcd for C_21_H_17_N_5_O_4_S_2_: C 53.75, H 3.55, N 14.76, S 13.43; Found: C 53.81, H 3.69, N 14.88, S 13.61. MS (EI, 60 eV): m/z (%): 467 (M^+^, 29).

#### *N*-(5-nitrothiazol-2-yl)-2-((4-oxo-3-propyl-3,4-dihydroquinazolin-2-yl)thio)acetamide (8i)

Light brown solid; isolated yield: 59% (238 mg), mp 179–181 °C; IR (KBr) υ: 3315, 3052, 2985, 2929, 1689, 1656, 1650, 1625, 1590, 1545, 1451, 1415, 1336, 1315, 1268, 1213, 1152, 980, 765, 741 cm^−1^. ^1^H NMR (300 MHz, DMSO-*d*_6_) δ 13.60 (s, 0.3H, exchangeable proton), 8.67 (s, 1H), 8.06 (dd, *J* = 8.0, 1.5 Hz, 1H), 7.79 (s, 0.1H, exchangeable proton), 7.74 (td, *J* = 8.2, 1.2 Hz, 1H), 7.43 (td, *J* = 8.2, 1.2 Hz, 1H), 7.325 (d, *J* = 8.2, 1H), 4.38 (s, 2H), 4.15–3.69 (m, 2H), 1.94–1.44 (m, 2H), 0.98 (t, *J* = 7.4 Hz, 3H). ^13^C NMR (76 MHz, DMSO) δ 169.57, 163.30, 160.83, 156.23, 146.96, 143.57, 141.83, 135.21, 126.96, 126.54, 125.96, 119.21, 46.27, 36.32, 21.48, 11.59. Anal.Calcd for C_16_H_15_N_5_O_4_S_2_: C 47.40, H 3.73, N 17.27, S 15.81; Found: C 47.61, H 4.02, N 17.16, S 16.11. MS (EI, 60 eV): m/z (%): 405 (M^+^, 28).

#### 2-((3-isopropyl-4-oxo-3,4-dihydroquinazolin-2-yl)thio)-*N*-(5-nitrothiazol-2-yl)acetamide (8j)

Yellow solid; isolated yield: 66% (267 mg), mp 165–167 °C; IR (KBr) υ: 3315, 3074, 2971, 2856, 1685, 1655, 1650, 1625, 1594, 1548, 1462, 1419, 1383, 1365, 1298, 1228, 1168, 1007, 759, 713 cm^−1^. ^1^H NMR (300 MHz, DMSO-*d*_6_) δ 8.47 (s, 1H), 8.05 (td, *J* = 8.2, 1.2 Hz, 1H), 7.76 (td, *J* = 8.2, 1.2 Hz, 1H), 7.51–7.31 (m, 2H), 4.80 (s, 1H), 4.23 (s, 2H), 1.63 (d, *J* = 6.7 Hz, 6H). ^13^C NMR (76 MHz, DMSO) δ 175.25, 174.05, 161.53, 157.39, 146.84, 146.67, 136.54, 134.92, 126.54, 126.10, 120.35, 52.72, 36.26, 19.66. Anal.Calcd for C_16_H_15_N_5_O_4_S_2_: C 47.40, H 3.73, N 17.27, S 15.81; Found: C 47.66, H 3.87, N 17.55, S 15.59. MS (EI, 60 eV): m/z (%): 405 (M^+^, 23).

#### 2-((3-butyl-4-oxo-3,4-dihydroquinazolin-2-yl)thio)-*N*-(5-nitrothiazol-2-yl)acetamide (8 k)

Brown solid; isolated yield: 68% (284 mg), mp 192–194 °C; IR (KBr) υ: 3310, 3042, 2996, 2921, 1687, 1658, 1651, 1628, 1592, 1547, 1456, 1412, 1333, 1309, 1274, 1223, 1175, 985, 760, 736 cm^−1^. ^1^H NMR (300 MHz, DMSO-*d*_6_) δ 8.59 (s, 1H), 7.97 (dd, *J* = 8.0, 1.5 Hz, 1H), 7.65 (td, *J* = 8.2, 1.2 Hz, 1H), 7.34 (td, *J* = 7.6, 1.2 Hz, 1H), 7.23 (d, *J* = 7.7 Hz, 1H), 4.31 (s, 2H), 3.99 (t, , *J* = 7.3 Hz, 2H), 1.72–1.57 (m, 2H), 1.37–1.29 (m, 2H), 0.88 (t, *J* = 7.3 Hz, 3H). ^13^C NMR (76 MHz, DMSO) δ 169.40, 163.01, 160.78, 156.14, 146.94, 143.46, 141.97, 135.16, 126.92, 126.52, 125.94, 119.19, 44.57, 36.22, 30.07, 20.11, 14.01. Anal.Calcd for C_17_H_17_N_5_O_4_S_2_: C 48.68, H 4.09, N 16.70, S 15.29; Found: C 48.48, H 3.92, N 16.44, S 15.52. MS (EI, 60 eV): m/z (%): 419 (M^+^, 22).

#### 2-((3-isobutyl-4-oxo-3,4-dihydroquinazolin-2-yl)thio)-*N*-(5-nitrothiazol-2-yl)acetamide (8 l)

Brown solid; isolated yield: 69% (285 mg), mp 183–185 °C; IR (KBr) υ: 3317, 3076, 2970, 2850, 1681, 1657, 1652, 1628, 1599, 1545, 1460, 1420, 1388, 1366, 1296, 1160, 1010, 762 cm^−1^. ^1^H NMR (400 MHz, DMSO-*d*_6_) δ 12.94 (s, 0.5 H, exchangeable proton), 8.66 (s, 1H), 8.05 (dd, *J* = 8.0, 1.5 Hz, 1H), 7.95 (s, 0.5 H, exchangeable proton), 7.73 (ddt, *J* = 7.2, 5.1, 1.6 Hz, 1H), 7.46–7.36 (m, 1H), 7.31 (d, *J* = 8.1 Hz, 1H), 4.36 (s, 2H), 3.94 (d, *J* = 7.4 Hz, 2H), 2.38–2.14 (m, 1H), 0.94 (d, *J* = 6.7 Hz, 6H). ^13^C NMR (101 MHz, DMSO) δ 169.62, 163.38, 161.17, 156.58, 146.84, 143.61, 141.74, 135.23, 127.07, 126.56, 125.94, 119.14, 51.21, 36.50, 27.92, 20.45, 20.43. Anal.Calcd for C_17_H_17_N_5_O_4_S_2_: C 48.68, H 4.09, N 16.70, S 15.29; Found: C 48.73, H 4.13, N 16.52, S 15.41. MS (EI, 60 eV): m/z (%): 419 (M^+^, 27).

#### 2-((3-allyl-4-oxo-3,4-dihydroquinazolin-2-yl)thio)-*N*-(5-nitrothiazol-2-yl)acetamide (8 m)

Brown solid; isolated yield: 74% (298 mg), mp 236–238 °C; IR (KBr) υ: 3322, 3065, 2921, 1688, 1650, 1651, 1628, 1591, 1551, 1492, 1487, 1452, 1410, 1348, 1310, 1285, 1225, 1171, 1080, 760, 730 cm^−1^. ^1^H NMR (300 MHz, DMSO-*d*_6_) δ 13.51 (s, 0.3H, Exchangeable proton), 8.67 (s, 1H), 8.07 (dd, *J* = 7.9, 1.5 Hz, 1H), 7.97 (s, 0.3H, Exchangeable proton), 7.76 (td, *J* = 8.0, 1.5 Hz 1H), 7.45 (td, *J* = 7.1, 1.5 Hz 1H), 7.34 (d, *J* = 8.1 Hz, 1H), 5.97 (ddt, *J* = 17.2, 10.4, 5.1 Hz, 1H), 5.32–5.09 (m, 2H), 4.75 (d, *J* = 5.1 Hz, 2H), 4.37 (s, 2H). ^13^C NMR (76 MHz, DMSO) δ 169.56, 163.35, 160.66, 156.45, 147.01, 143.58, 141.81, 135.33, 131.76, 127.03, 126.62, 126.03, 119.16, 118.25, 46.54, 36.41. Anal.Calcd for C_16_H_13_N_5_O_4_S_2_: C 47.64, H 3.25, N 17.36, S 15.89; Found: C 47.86, H 3.16, N 17.41, S 15.76. MS (EI, 60 eV): m/z (%): 403 (M^+^, 26).

#### 2-((3-cyclopentyl-4-oxo-3,4-dihydroquinazolin-2-yl)thio)-*N*-(5-nitrothiazol-2-yl)acetamide (8n)

Brown solid; isolated yield: 70% (301 mg), mp 200–202 °C; IR (KBr) υ: 3329, 3062, 2929, 2893, 1686, 1652, 1645, 1620, 1591, 1551, 1478, 1424, 1388, 1341, 1291, 1245, 1153, 1012, 763, 692 cm^−1^. ^1^H NMR (300 MHz, DMSO-*d*_6_) δ 8.48 (s, 1H), 8.05 (dd, *J* = 7.9, 1.5 Hz, 1H), 7.75 (td, *J* = 8.2, 1.2 Hz, 1H), 7.52–7.30 (m, 2H), 5.03–4.77 (m, 1H), 4.24 (s, 2H), 2.26–2.19 (m, 2H), 2.06–1.82 (m, 5H), 1.74–1.46 (m, 2H). ^13^C NMR (76 MHz, DMSO) δ 175.06, 173.66, 161.02, 157.82, 146.77, 146.53, 136.77, 134.93, 126.58, 126.16, 126.11, 120.19, 60.10, 36.27, 28.74, 26.09. Anal.Calcd for C_18_H_17_N_5_O_4_S_2_: C 50.11, H 3.97, N 16.23, S 14.86; Found: C 49.89, H 4.16, N 16.34, S 14.59. MS (EI, 60 eV): m/z (%): 431 (M^+^, 29).

### Urease inhibitory activity

Urease inhibition effects of the synthesized compounds were determined according to the previously reported procedure^47–49^. 100 μL of the synthesized compounds at different concentrations was added to 850 μL of urea as substrate and 15 μL urease (0.135 units dissolved in PBS, pH 7.4). After 30 min, to 100 μL of the incubated solution, 500 μL solution I (5.0 g phenol and 25.0 mg sodium nitroprusside in 500 mL water) was added followed by the addition of 500 μL of solution II (2.5 g sodium hydroxide, 4.2 mL sodium hypochlorite, and 5% chlorine in 500 mL water) which was further incubated at 37 °C for 30 min. The absorbance was determined by measuring indophenols at 625 nm. Thiourea was used as the standard inhibitor for urease. The IC_50_ values for all synthesized compounds were calculated using GraphPad Prism software (GraphPad Software, Inc., San Diego, CA).

### Kinetic studies

The kinetic study for the inhibition of urease by compound **8h** was carried out using four different concentrations of inhibitor. For the kinetic study of urease, compound **8h** was used at the concentrations of 0, 1, 2, and 4 μM. The Lineweaver–Burk reciprocal plot was constructed by plotting 1/V against 1/[S] at variable concentrations of the substrate urea (3.12–100 mM). The inhibition constant K_i_ was calculated by the plot of slopes versus the corresponding concentrations of the compound **8h**.

### Molecular docking procedure

To perform the molecular modeling investigations, the Maestro Molecular Modeling platform (version 10.5) by Schrödinger, LLC has been used^50,52^. The X-ray crystallographic structure of the jack bean urease in complex with acetohydroxamic acid was downloaded from the protein data bank (www.rcsb.com) by the PDB ID: 4h9m. The protein is then prepared using a protein preparation wizard^51^. At this point, all water molecules and co-crystallised ligands were removed, the missing side chains and loops were filled using the prime tool^54^, and PROPKA assigned H-bonds at pH: 7.4. In order to prepare the ligands, the 2D structures of the ligands were drawn in ChemDraw (ver. 16) and converted into SDF files, which were used further by the ligprep module^52^. The ligand was prepared by OPLS_2005 force field using EPIK at a target pH of 7.0 ± 2^53^.

To gain a better understanding of the active site residue conformational change in the [ES] complex, the induced fit docking method was utilized for docking the urea in the active site of the molecule^54^. AHA was considered as the grid center and the maximum number of 20 poses was calculated with receptor and ligand van der Waals radii of 0.7 A and 0.5 A, respectively. Structures with prime energy levels beyond 30 kcal/mol were eliminated based on standard precious glide docking. The Site map tool was used to find the possible allosteric binding sites of the [ES] complex^55^. The site map was tasked to report up to 5 potential binding sites with at least 15 site points per each reported site by more restrictive definition of hydrophobicity. The grid box was generated for each binding site using entries with a box size of 25 A, compound **8h** was docked on binding sites using glide with extra precision and flexible ligand sampling, reporting 10 poses per ligand to form the final [ESI] complex^56^.

### Antimicrobial activity against ureolytic microorganisms

The antimicrobial activity of compounds against the microorganisms including *C. neoformans* (H99), and clinical isolate of *P.vulgaris* was assessed using the microbroth dilution method, as recommended by the Clinical and Laboratory Standards Institute (CLSI) (M07-A9 for bacteria; M27-A3 for yeasts). The compounds were diluted, and stock solutions of 20 mg/ml in DMSO were prepared. Mueller–Hinton Broth (HiMedia) and RPMI-1640 (Sigma) were prepared as recommended for antimicrobial susceptibility testing of bacterial and fungal strains, respectively. Two-fold dilutions were made in the range of 1–512 μg/ml for tested compounds. The microbroth dilution test was accomplished using a 96-well microtiter plate, containing growth control (yeast culture in broth media) and sterility control (broth media without fungal culture). The antimicrobial susceptibility test was accomplished by adding a cell suspension adjusted to the 0.5 McFarland standard (1–2 × 10^8^ CFU/mL for bacterial strains; 1−5 × 10^6^ cells/ml for yeast) to different concentrations of tested compounds. Following incubation, the minimum inhibitory concentration (MIC) was established as the lowest concentration of compound that completely inhibits the growth of the organism in wells as detected visually. All experiments were performed in duplicates.

### Anti-ureolytic activity against ureolytic microorganisms

The colorimetric microdilution technique using urea broth media (Merck, supplemented with glucose; pH = 6 for *C. neoformans*) was used to examine the urolytic activity of *C. neoformans* (H99), and clinical isolate of *P.vulgaris* treated with tested substances. Compounds in the concentration range of 1–512 μg/mL were exposed to ureolytic microorganisms, and the color of the medium was evaluated visually and spectroscopically at 560 nm after three days for *C. neoformans* and 24 h for *P. vulgaris*. The positive control, which included ureolytic bacteria but no drugs, changed color from yellow to dark pink or magenta. This shifts, allowing the determination of the inhibitory activity of compounds against urease activity of organisms even without a microtiter plate reader^57,58^.

### In silico pharmacokinetic properties of synthesized compounds

SwissADME and pkCSM servers were used to determine the physicochemical and drug-likeness properties of the derivatives.

## Supplementary Information

Supplementary Information.

## Data Availability

The datasets used and analyzed during the current study are available from the corresponding author on reasonable request.
