# Supplementary material for: Design and synthesis of novel nitrothiazolacetamide conjugated to different thioquinazolinone derivatives as anti-urease agents
Source: Sci Rep. 2022 Feb 7;12:2003. doi: 10.1038/s41598-022-05736-4 (PMC8821706; doi:10.1038/s41598-022-05736-4)

**
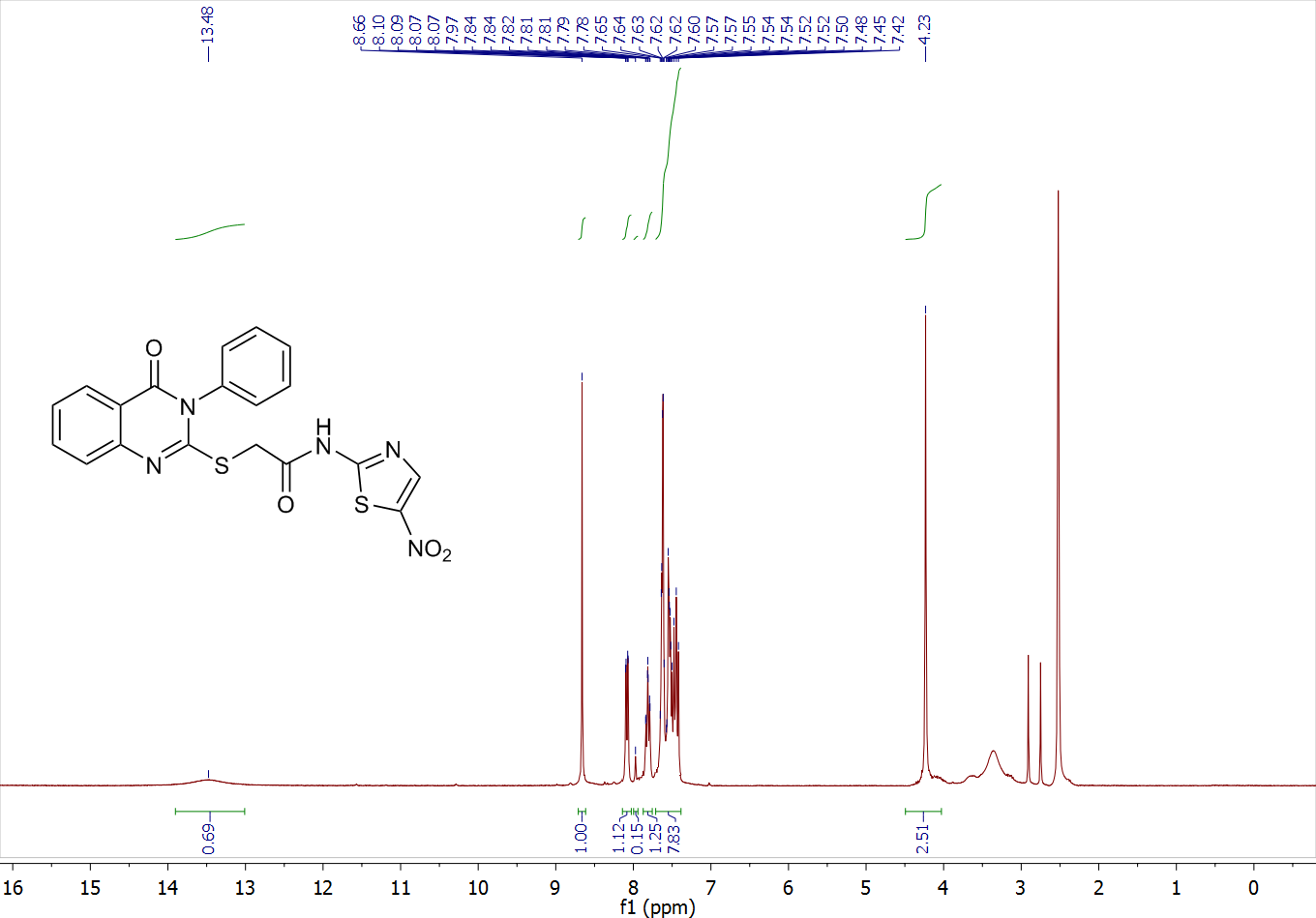
*N*-(5-nitrothiazol-2-yl)-2-((4-oxo-3-phenyl-3,4-dihydroquinazolin-2-yl)thio)acetamide (8a)**

**
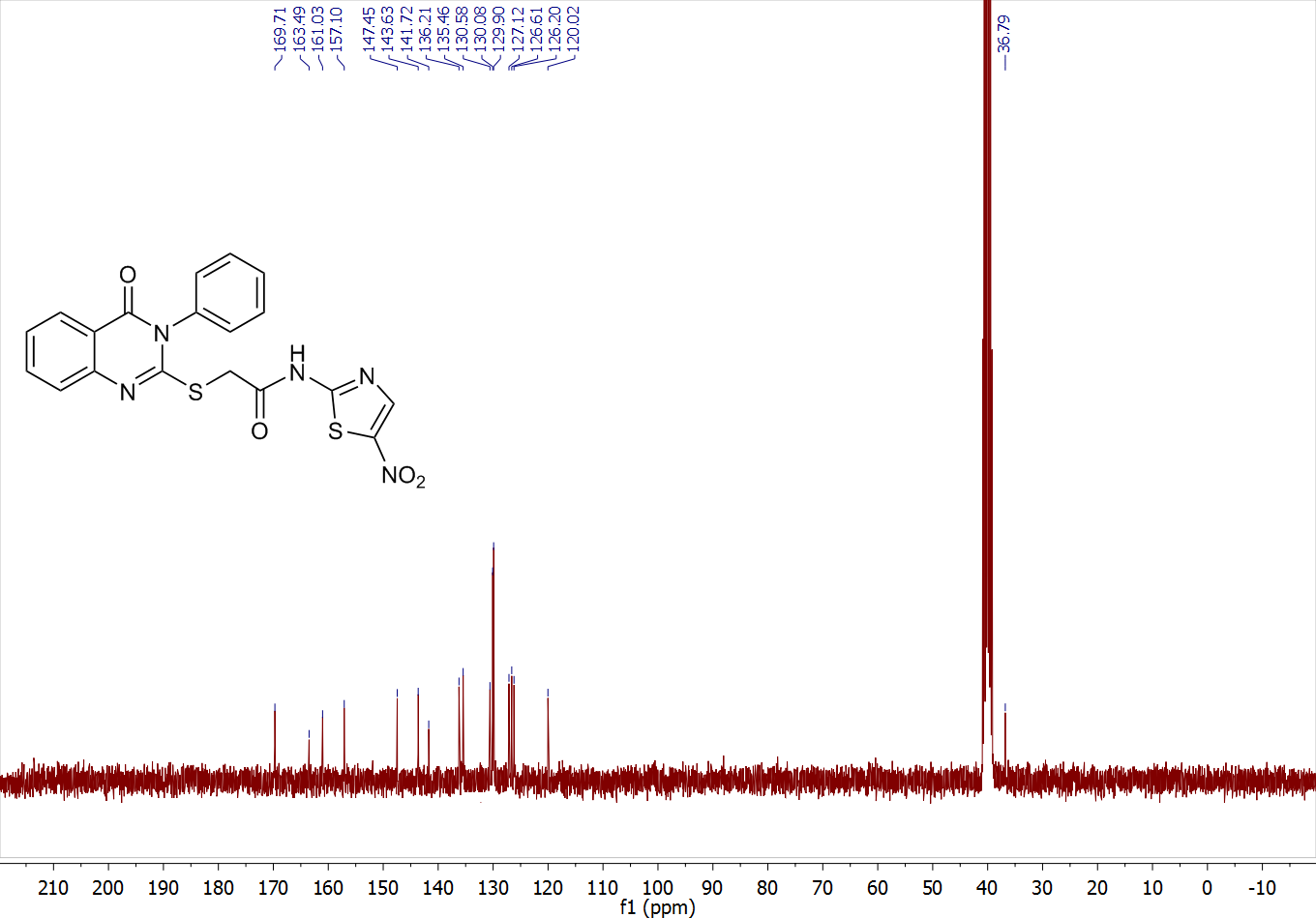
**

**
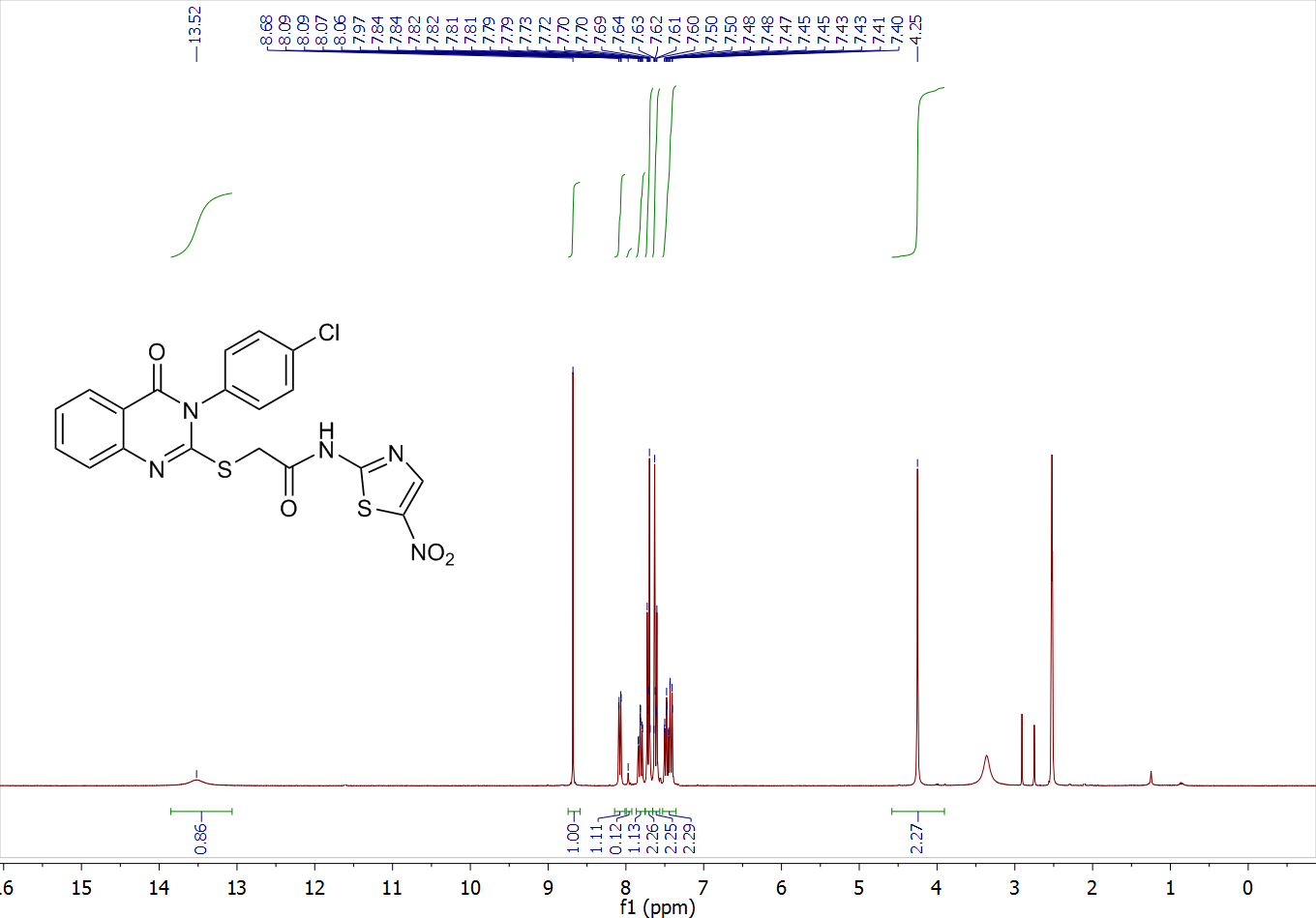
**

**
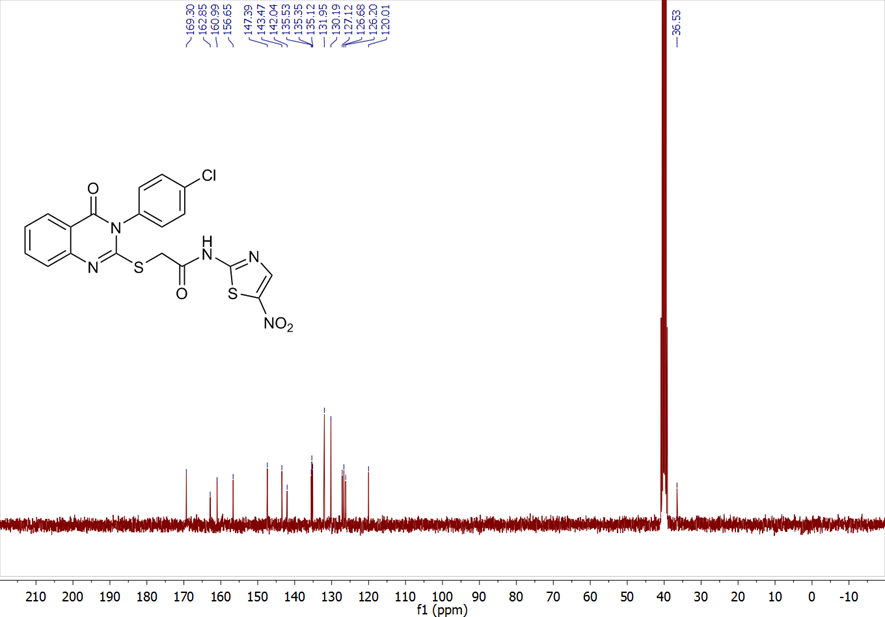
**

**2-((3-(4-bromophenyl)-4-oxo-3,4-dihydroquinazolin-2-yl)thio)-*N*-(5-nitrothiazol-2-yl)acetamide (8c)**

**
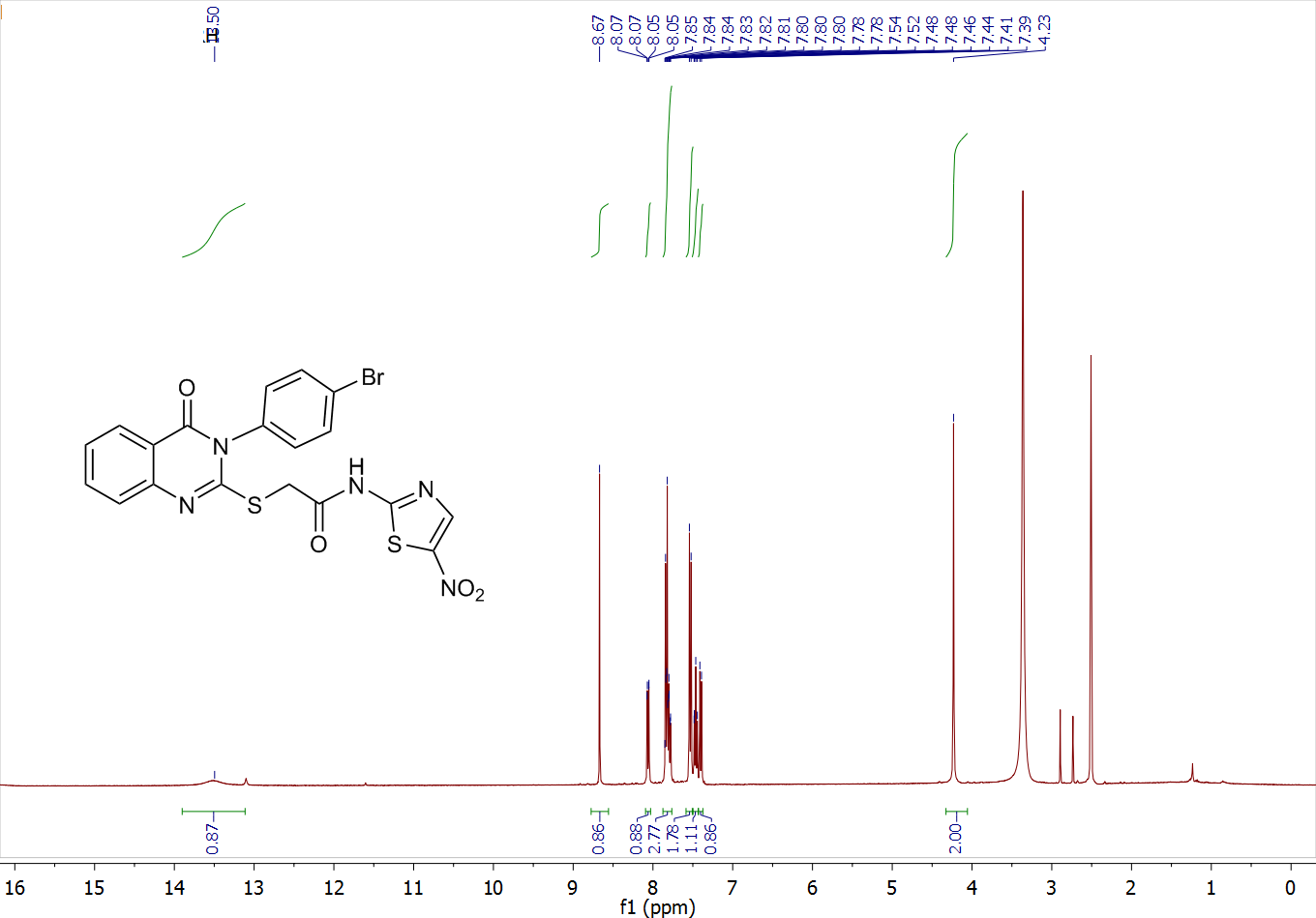
**


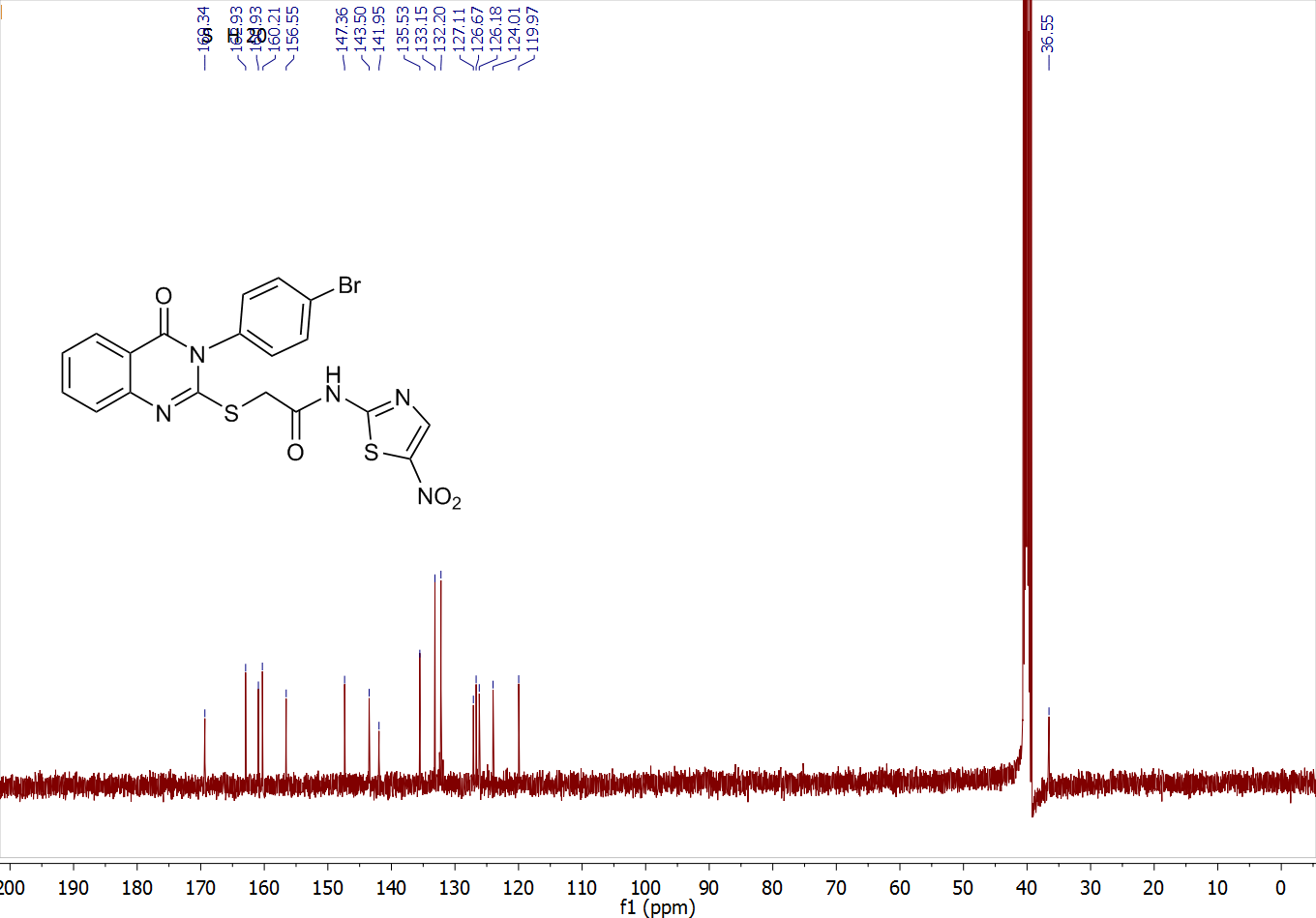


**2-((3-(4-methoxyphenyl)-4-oxo-3,4-dihydroquinazolin-2-yl)thio)-*N*-(5-nitrothiazol-2-yl)acetamide (8d)**

**
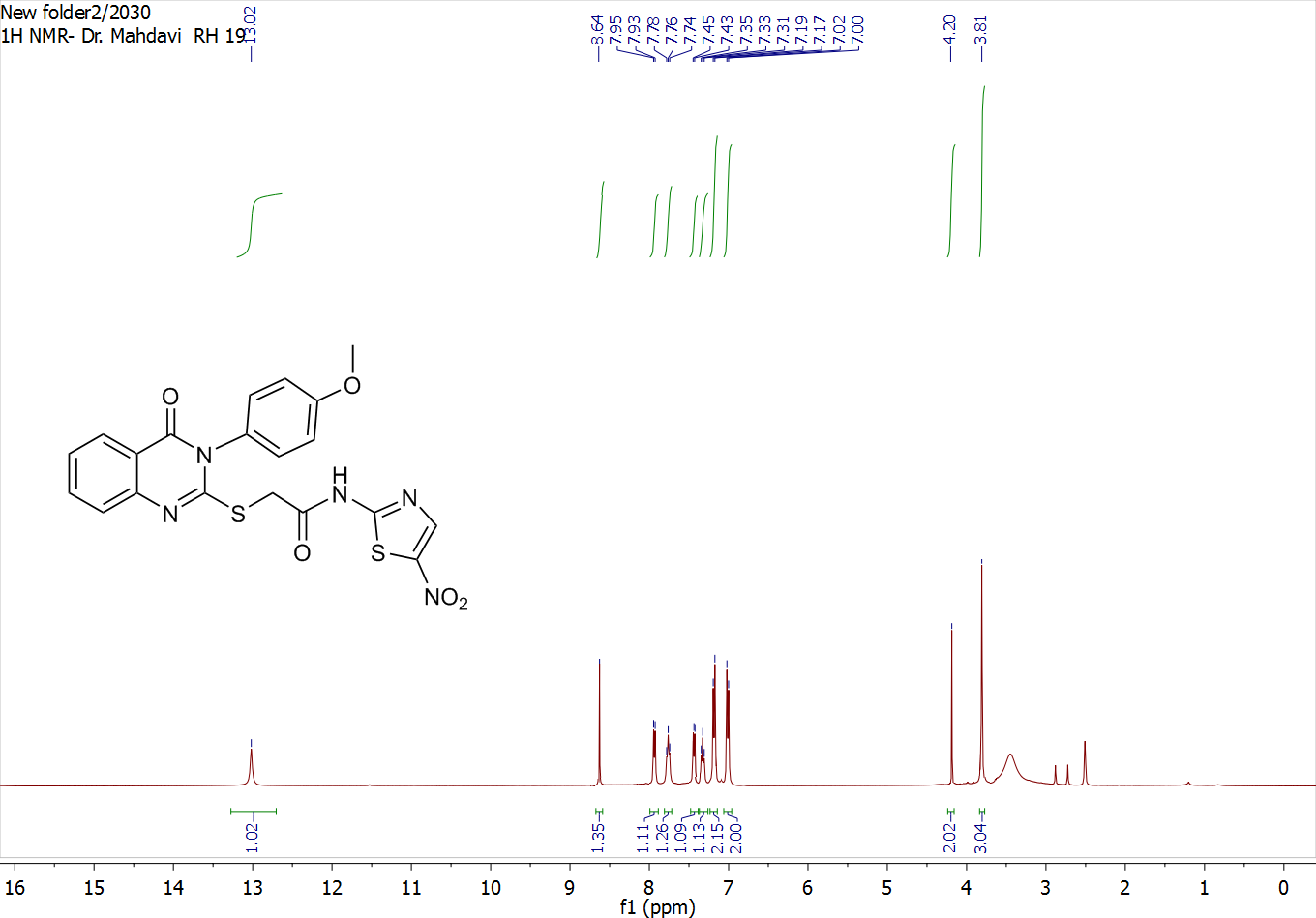
**

**
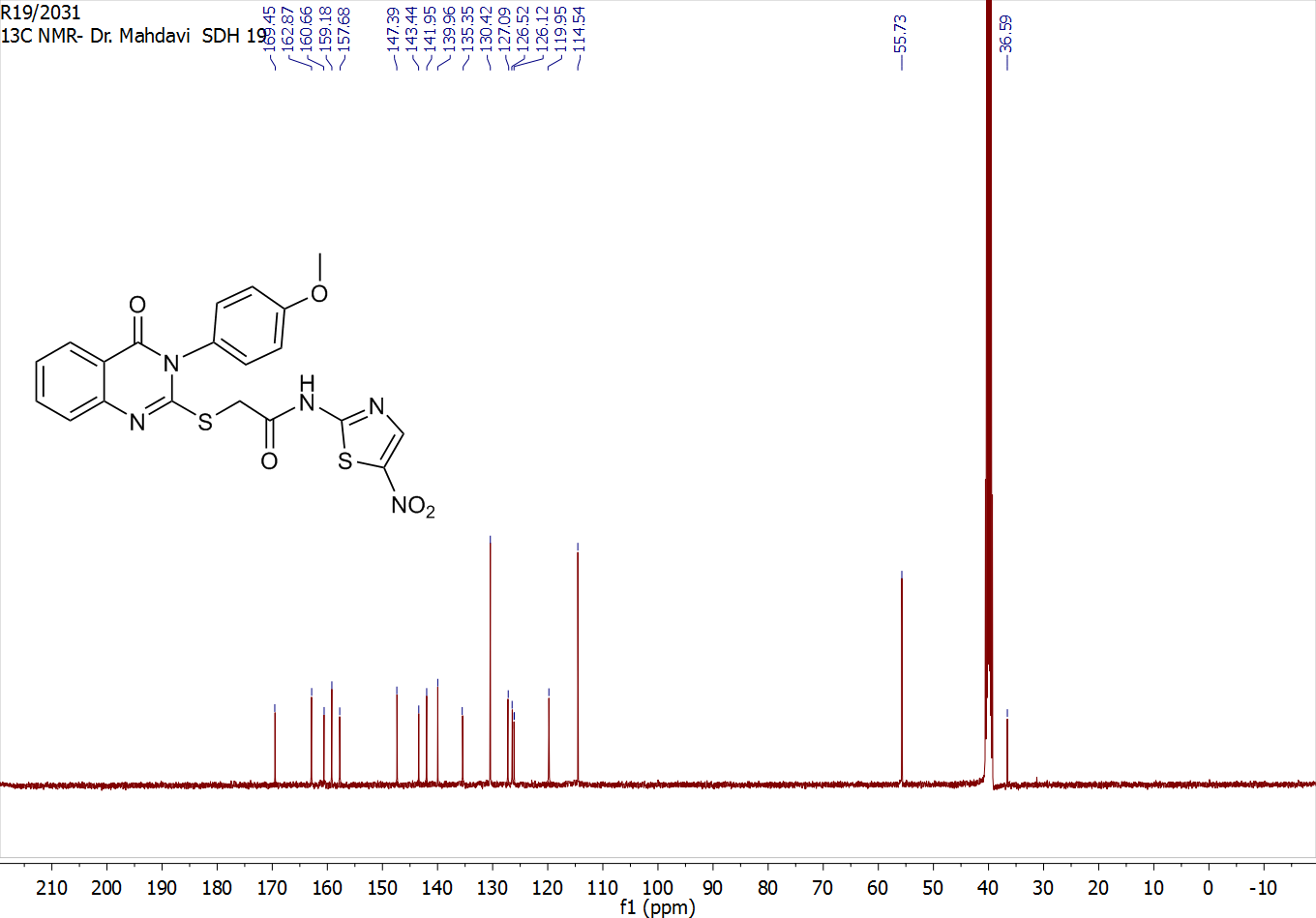
**


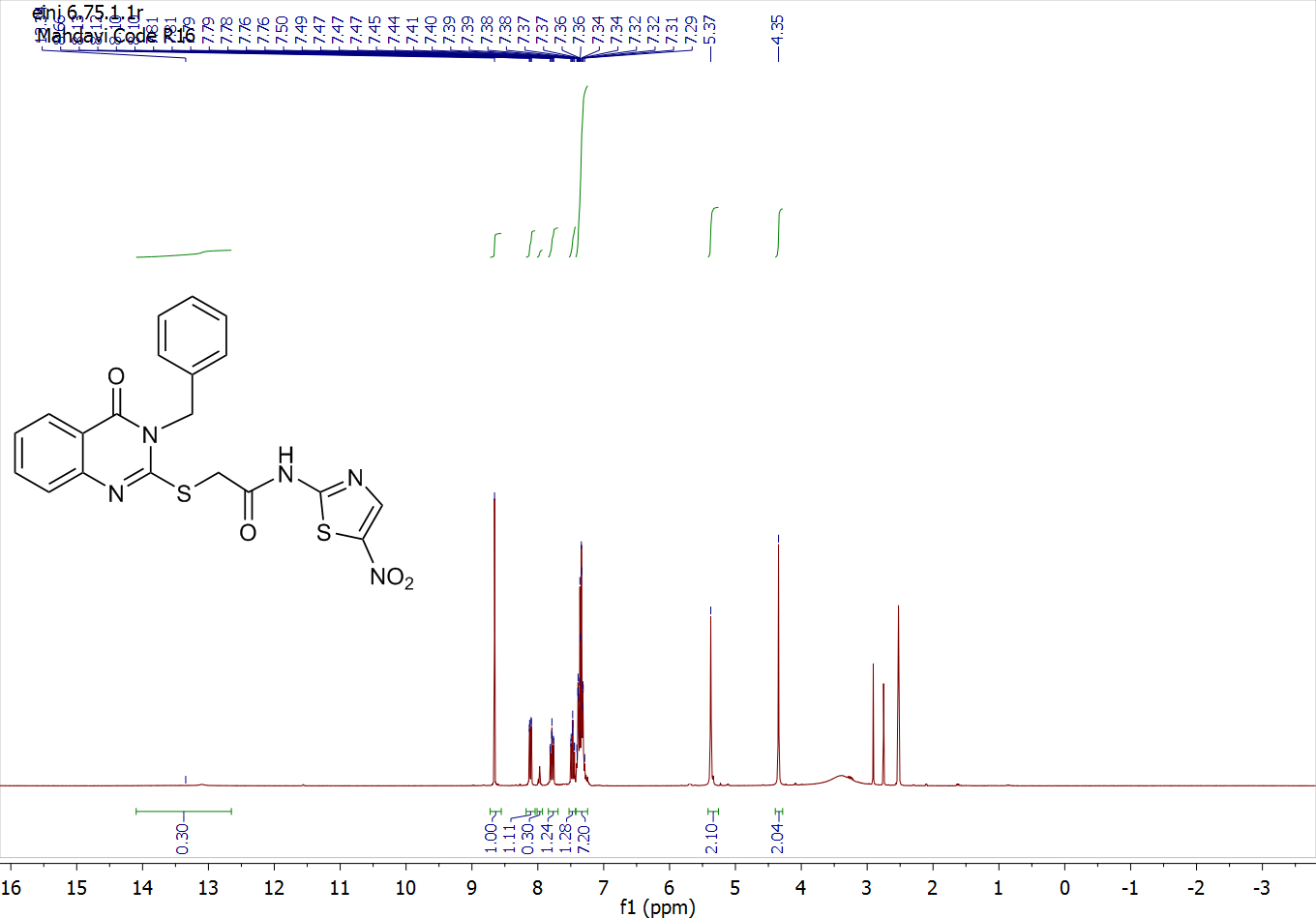
**2-((3-benzyl-4-oxo-3,4-dihydroquinazolin-2-yl)thio)-*N*-(5-nitrothiazol-2-yl)acetamide (8e)**


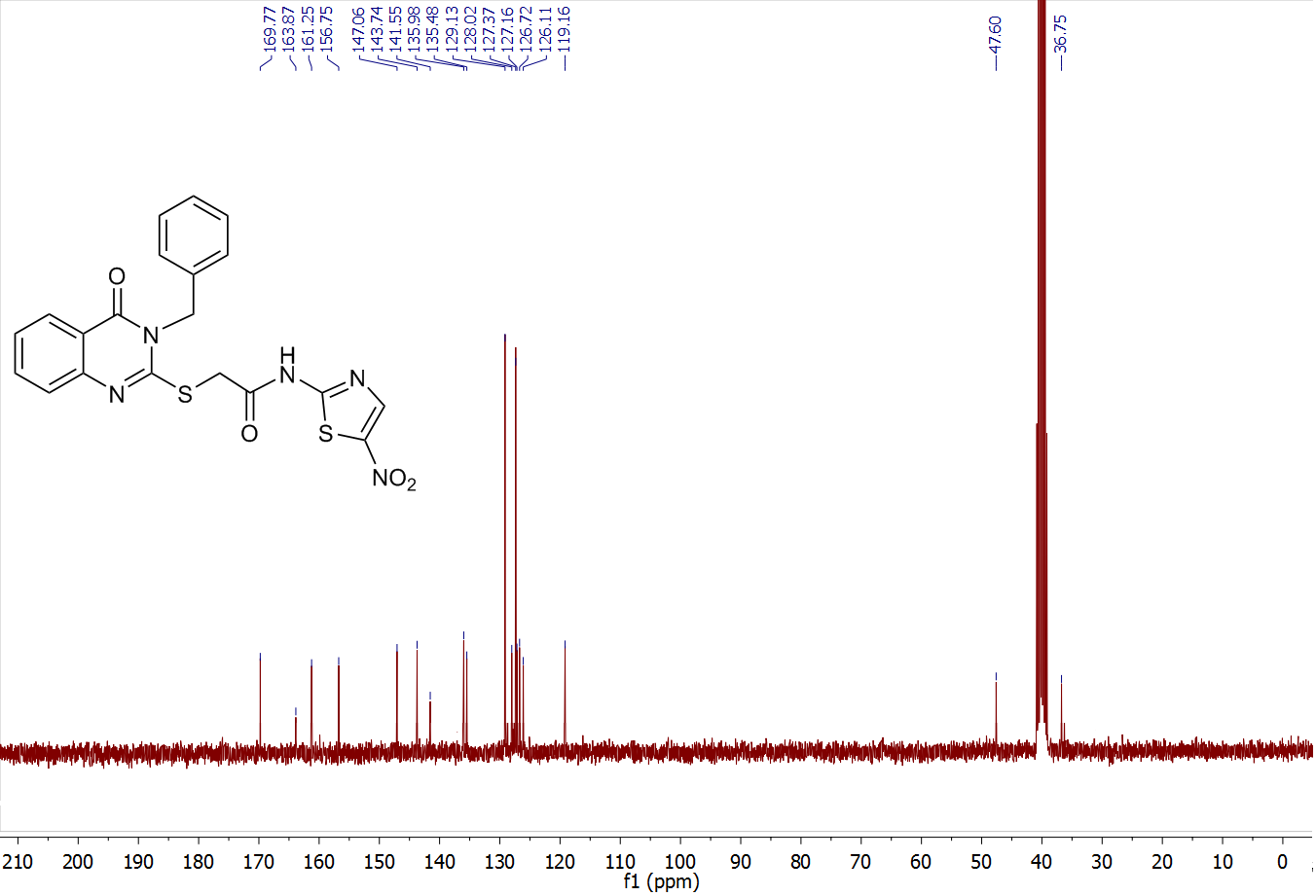


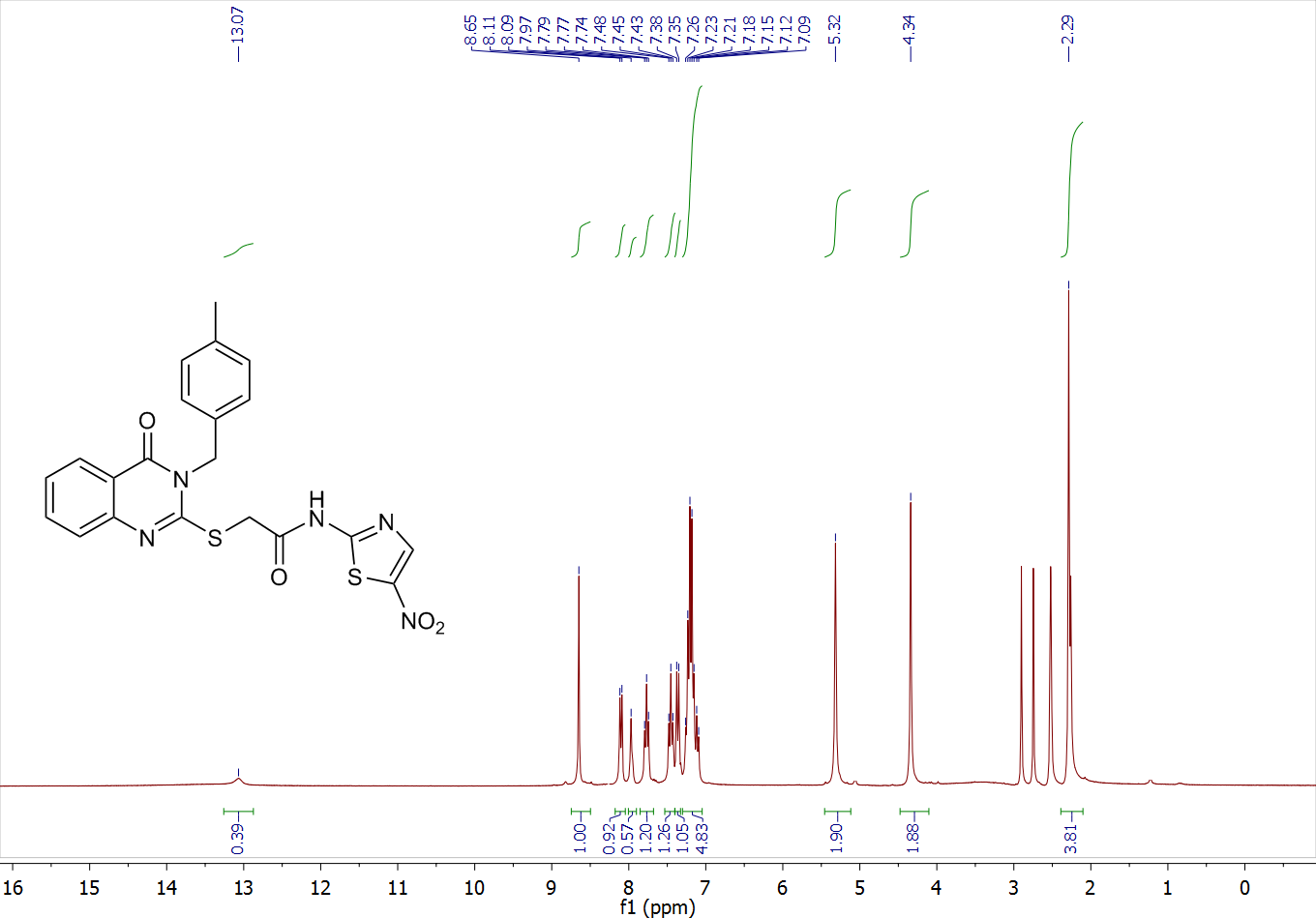
**2-((3-(4-methylbenzyl)-4-oxo-3,4-dihydroquinazolin-2-yl)thio)-*N*-(5-nitrothiazol-2-yl)acetamide (8f)**


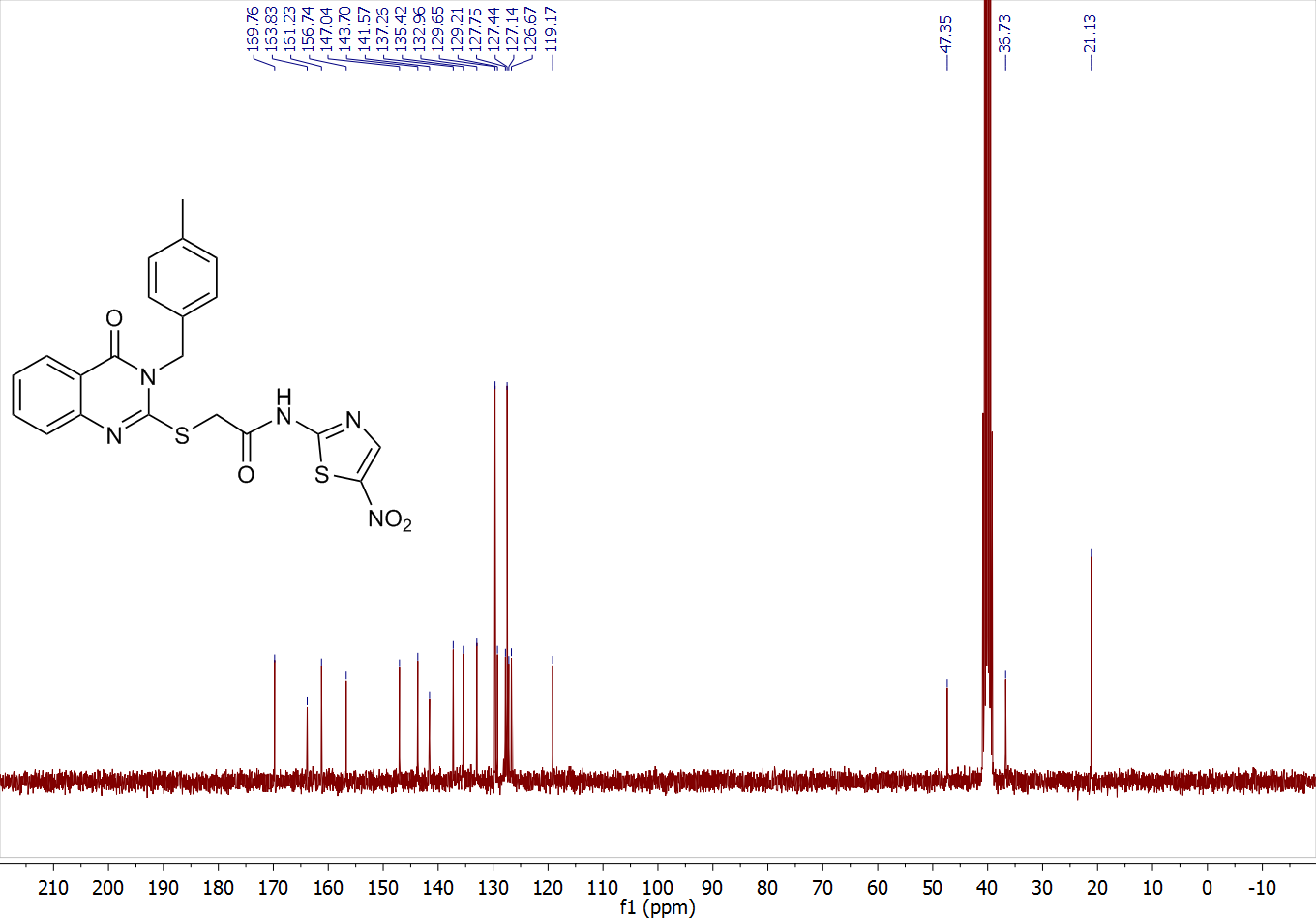


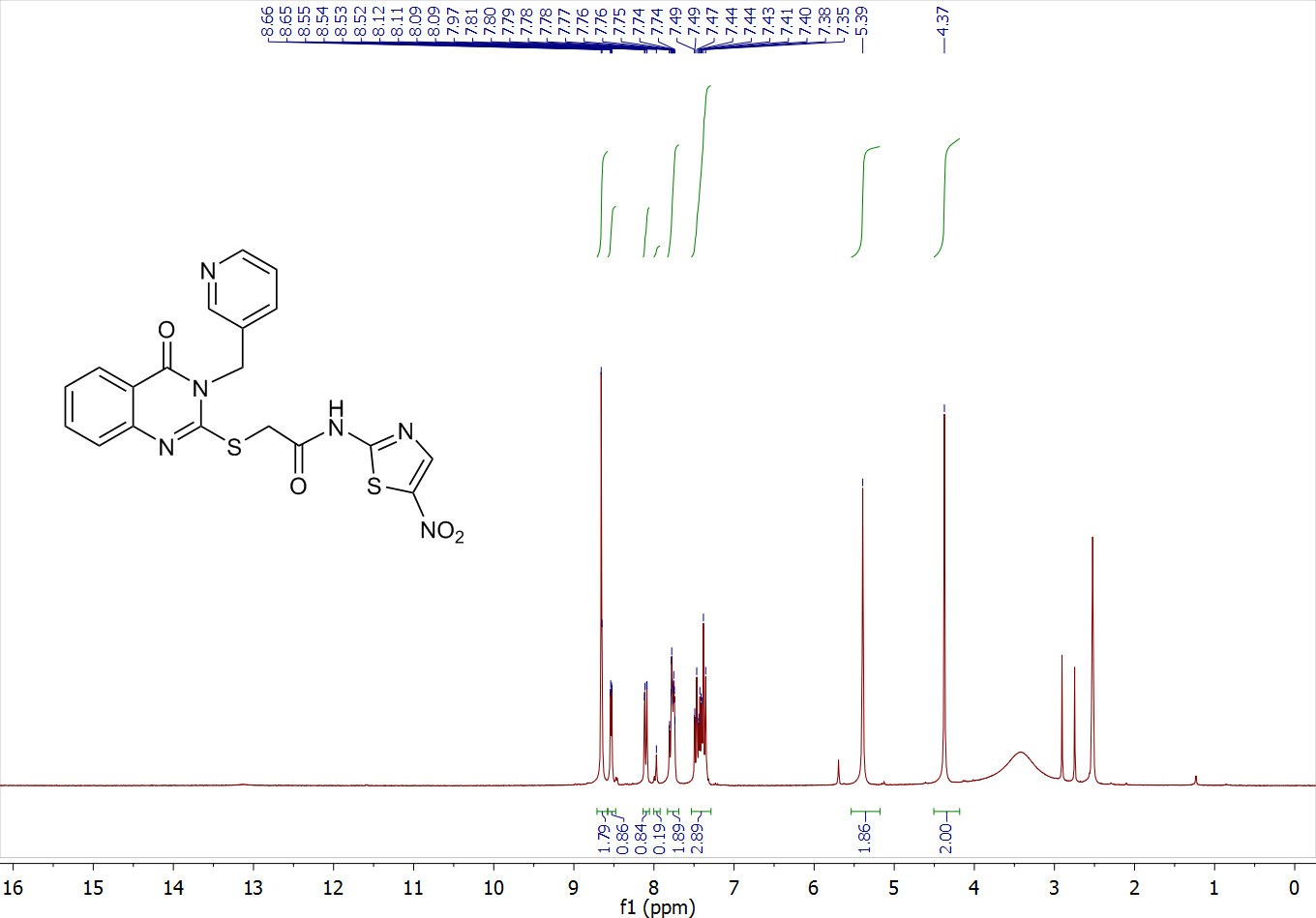
***N*-(5-nitrothiazol-2-yl)-2-((4-oxo-3-(pyridin-3-ylmethyl)-3,4-dihydroquinazolin-2-yl)thio)acetamide (8g)**


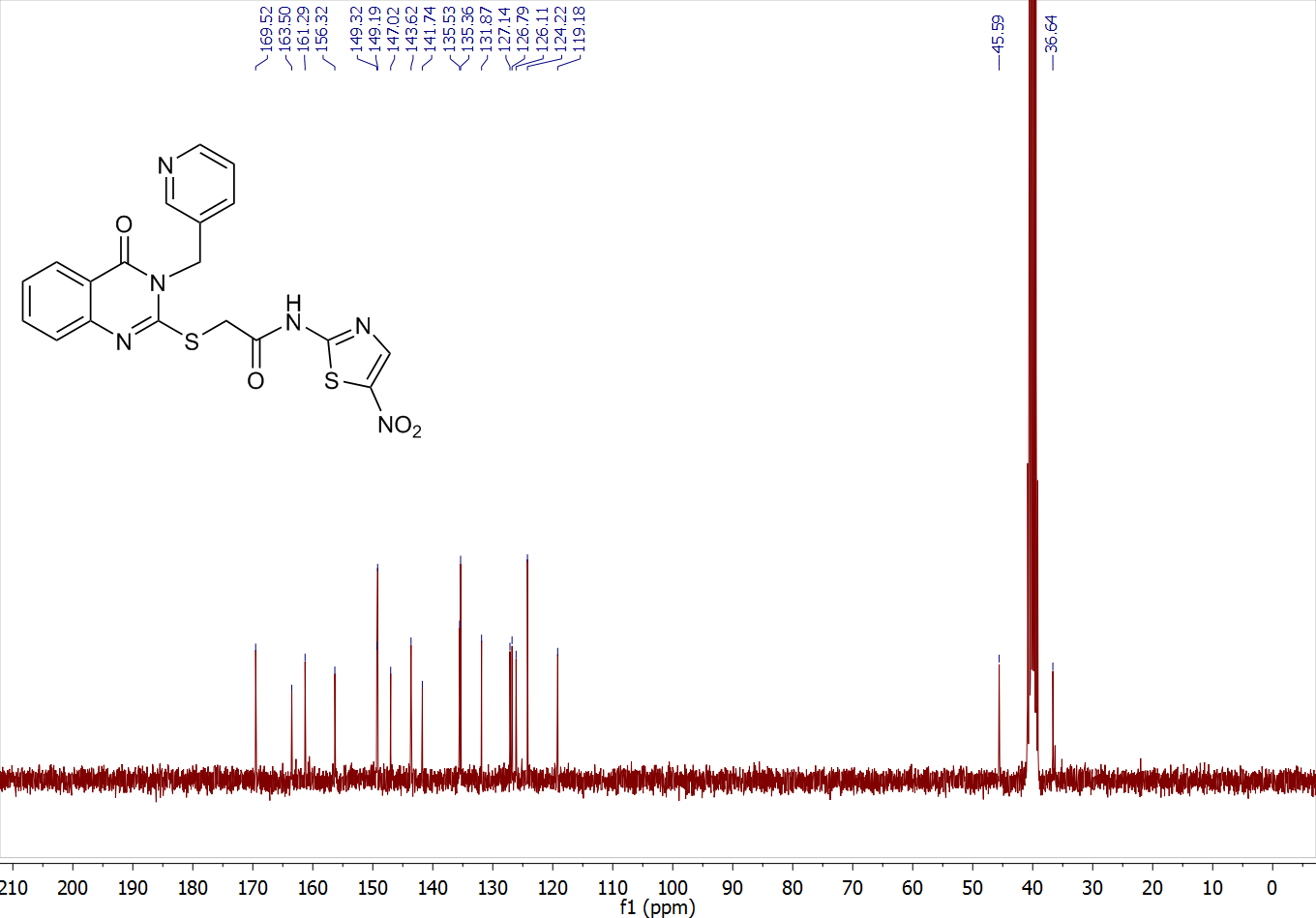


***N*-(5-nitrothiazol-2-yl)-2-((4-oxo-3-phenethyl-3,4-dihydroquinazolin-2-yl)thio)acetamide (8h)**


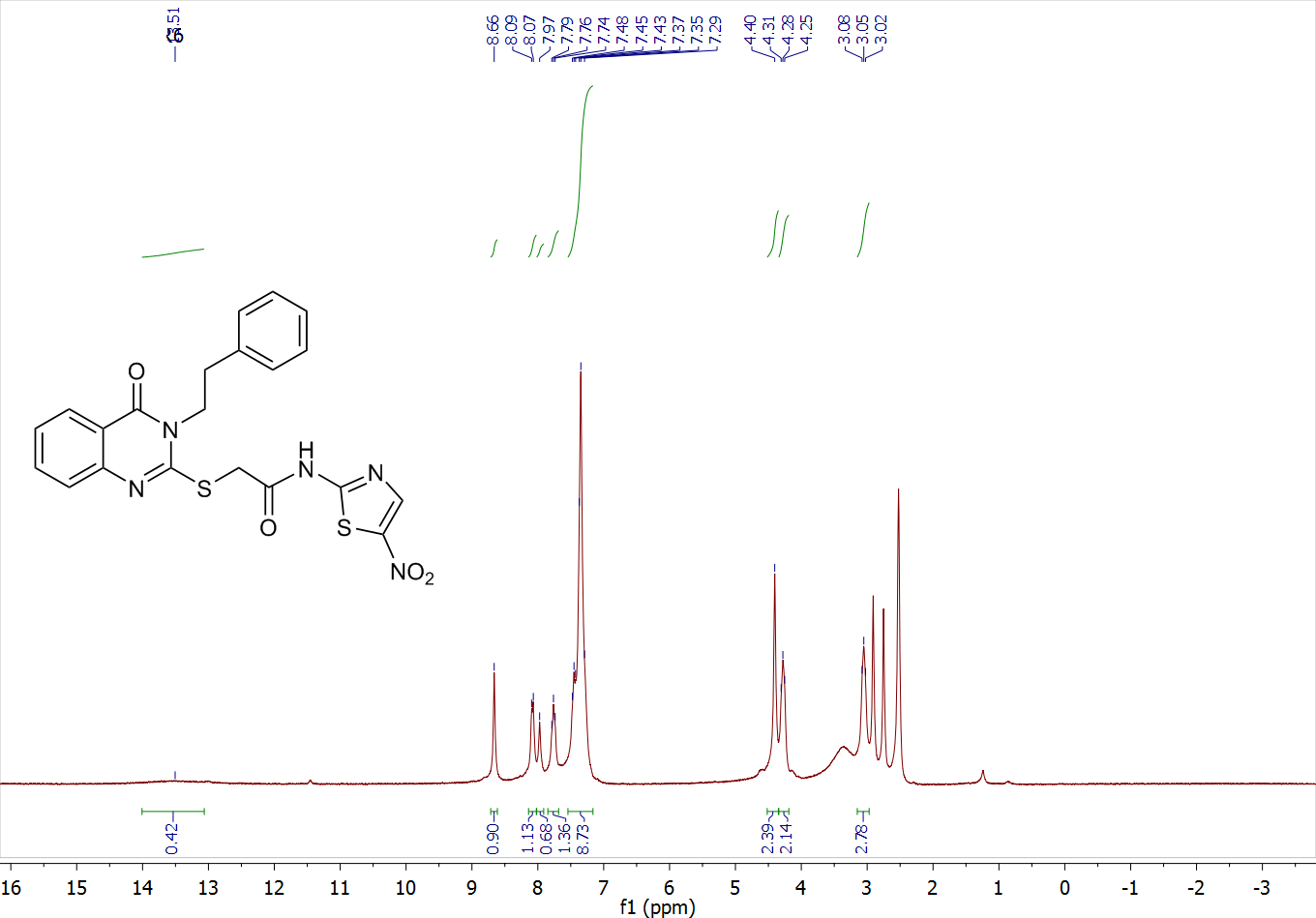


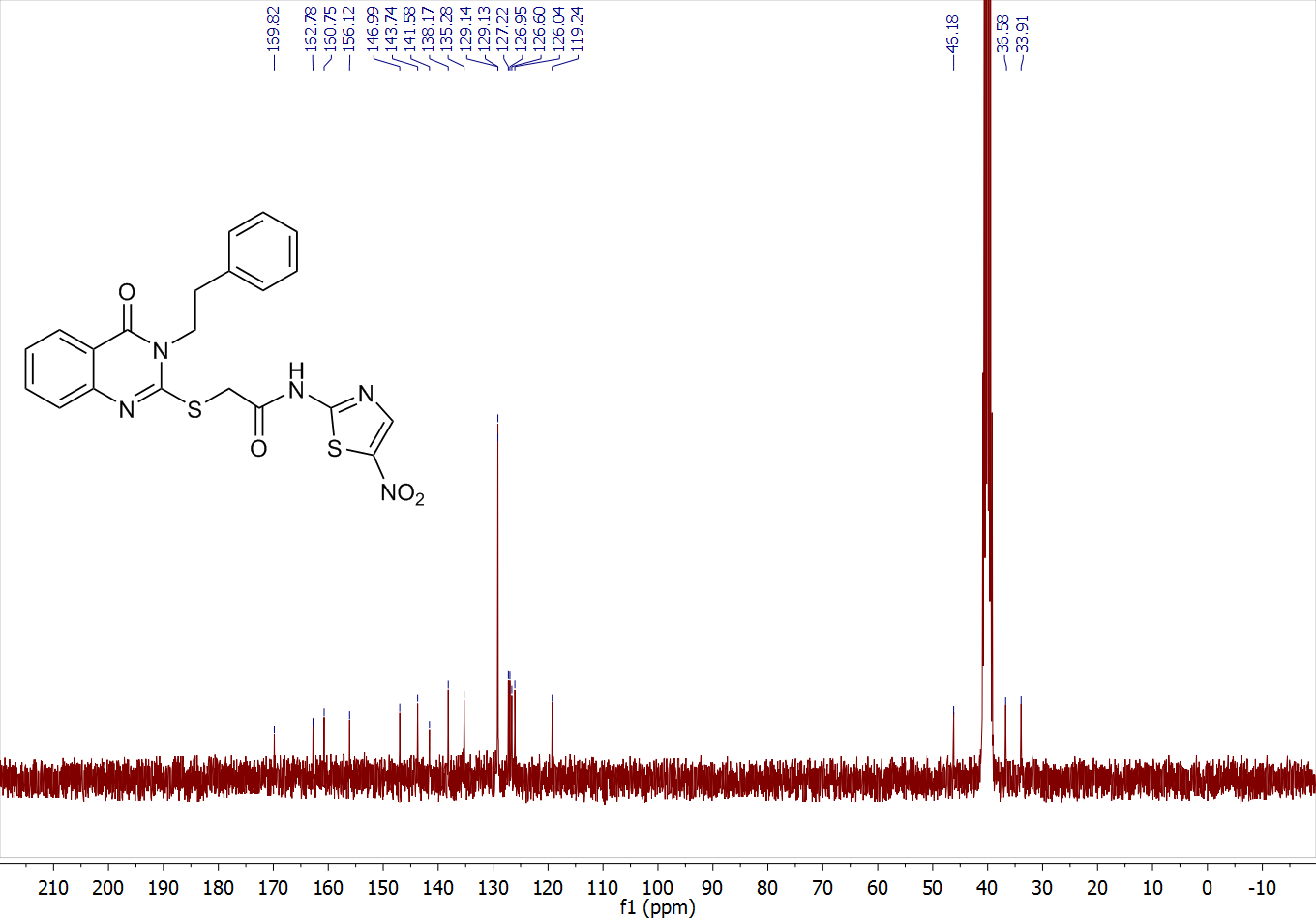


***N*-(5-nitrothiazol-2-yl)-2-((4-oxo-3-propyl-3,4-dihydroquinazolin-2-yl)thio)acetamide (8i)**


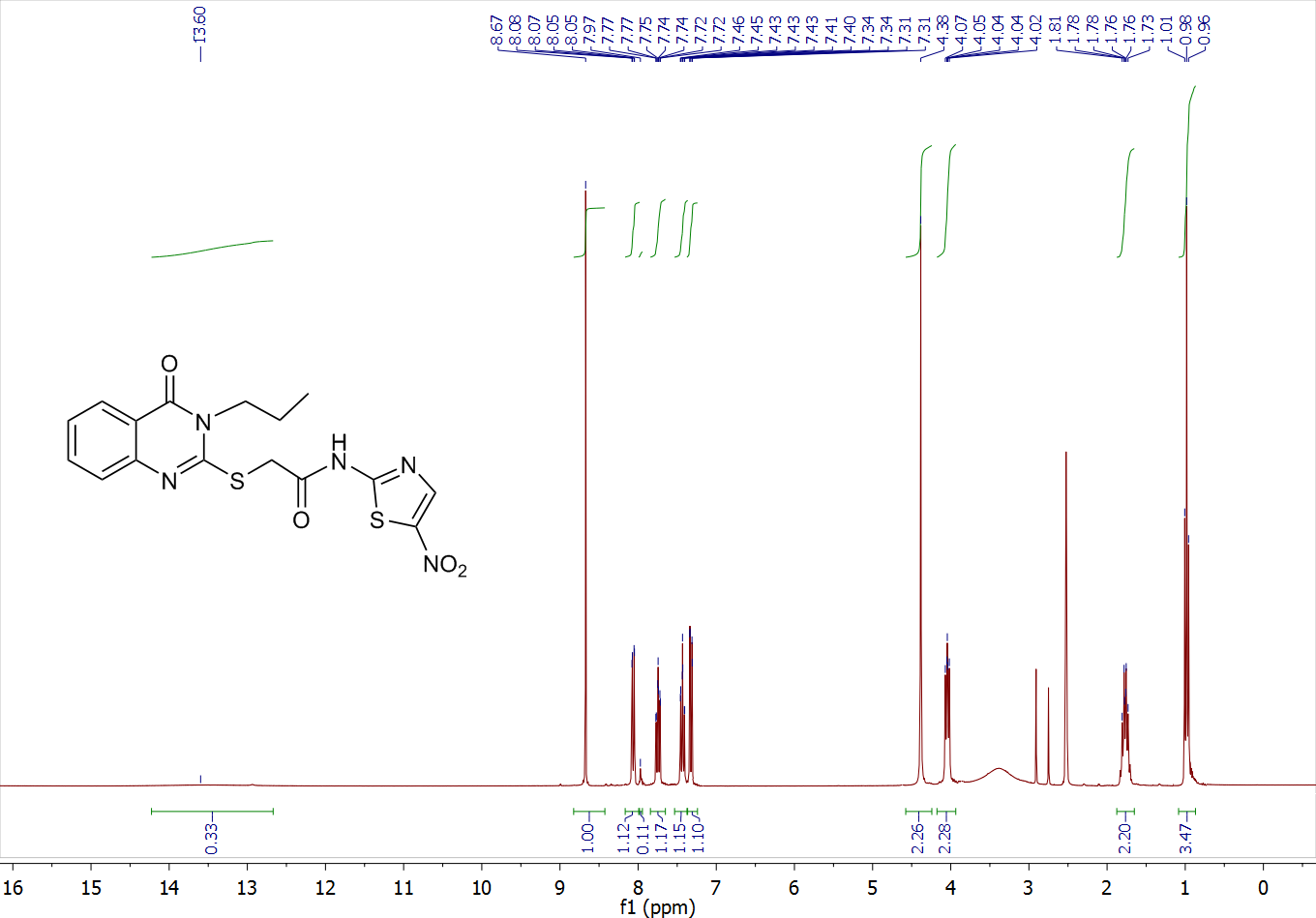


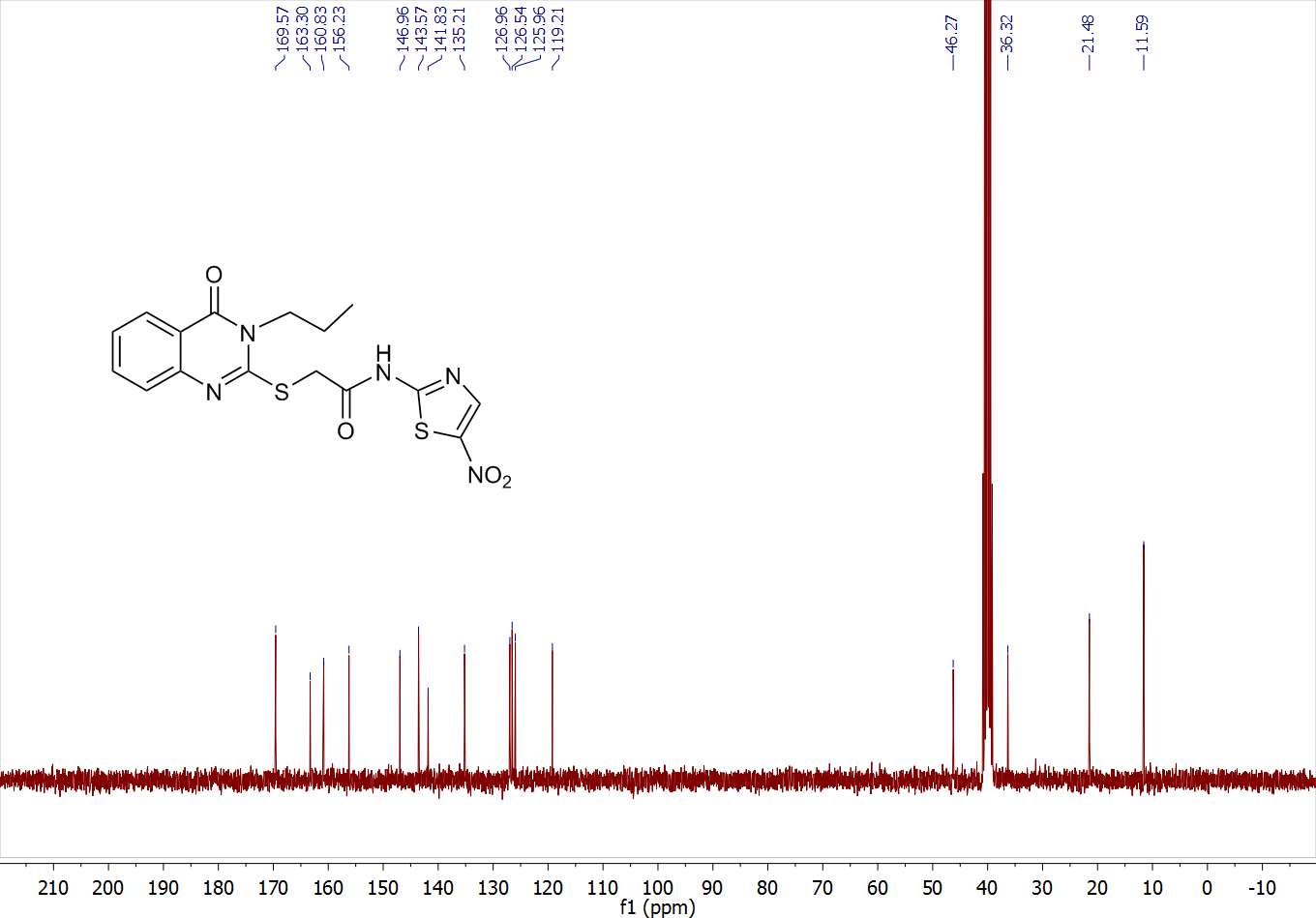


**2-((3-isopropyl-4-oxo-3,4-dihydroquinazolin-2-yl)thio)-*N*-(5-nitrothiazol-2-yl)acetamide (8j)**


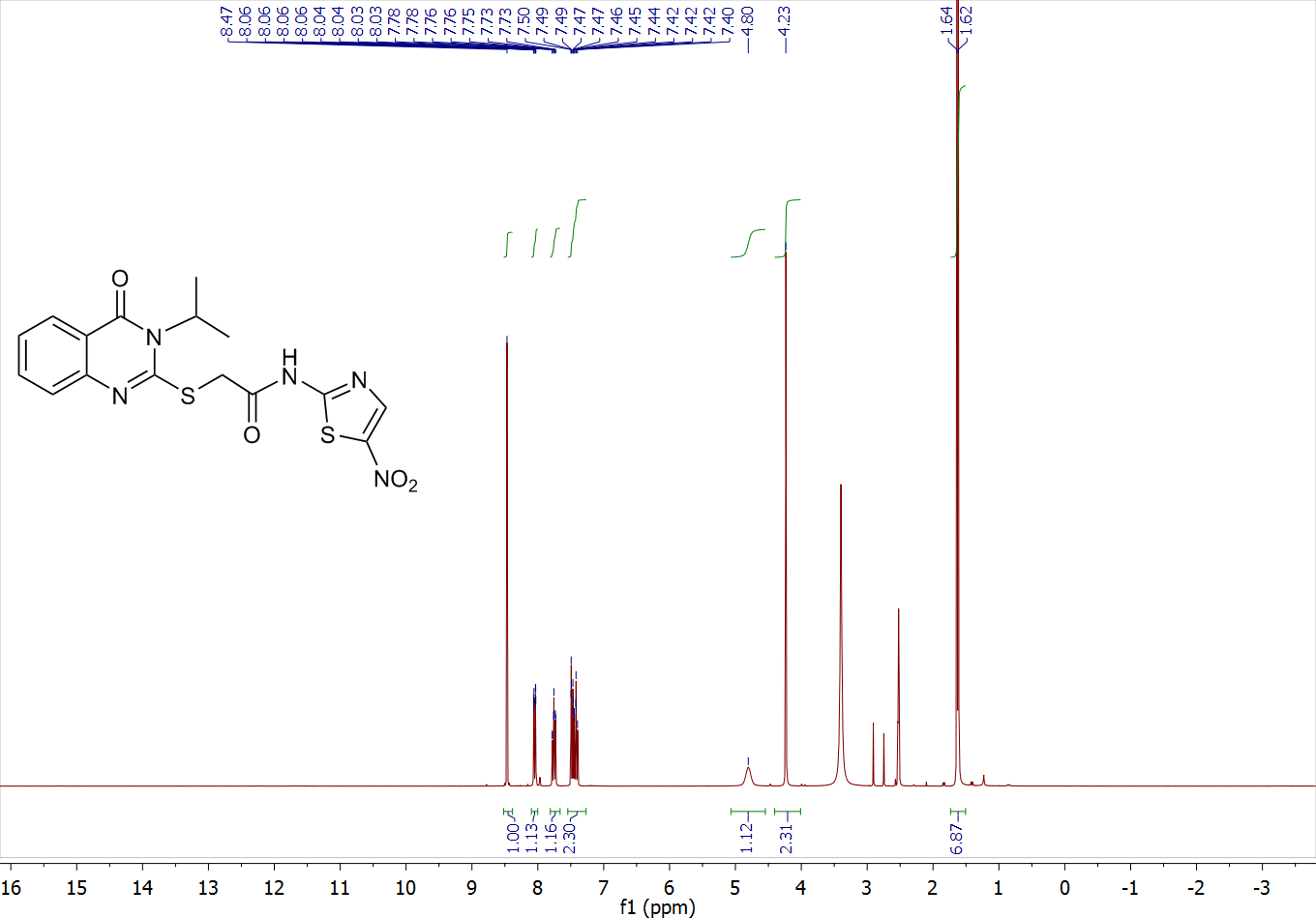


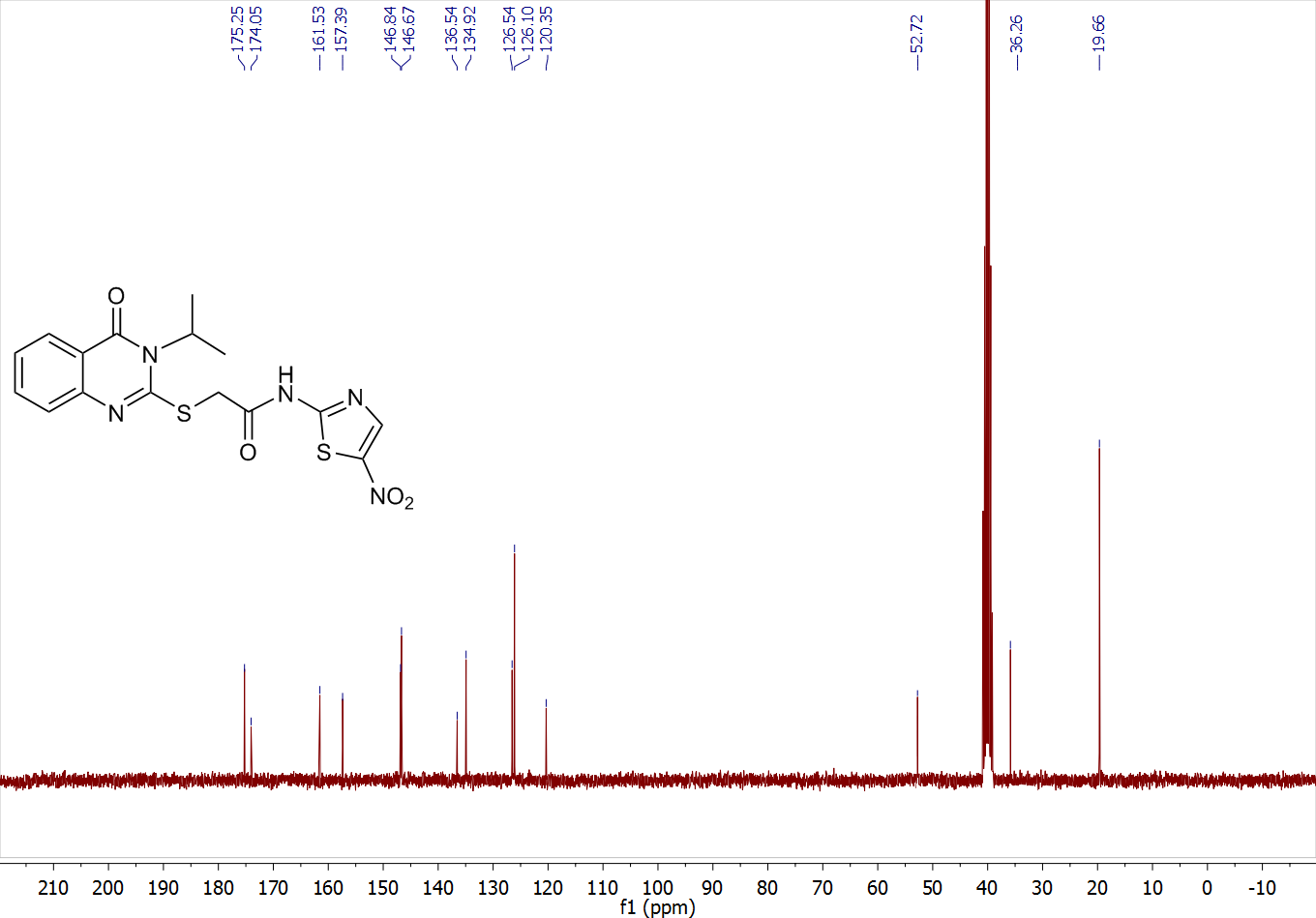


**2-((3-butyl-4-oxo-3,4-dihydroquinazolin-2-yl)thio)-*N*-(5-nitrothiazol-2-yl)acetamide (8k)**


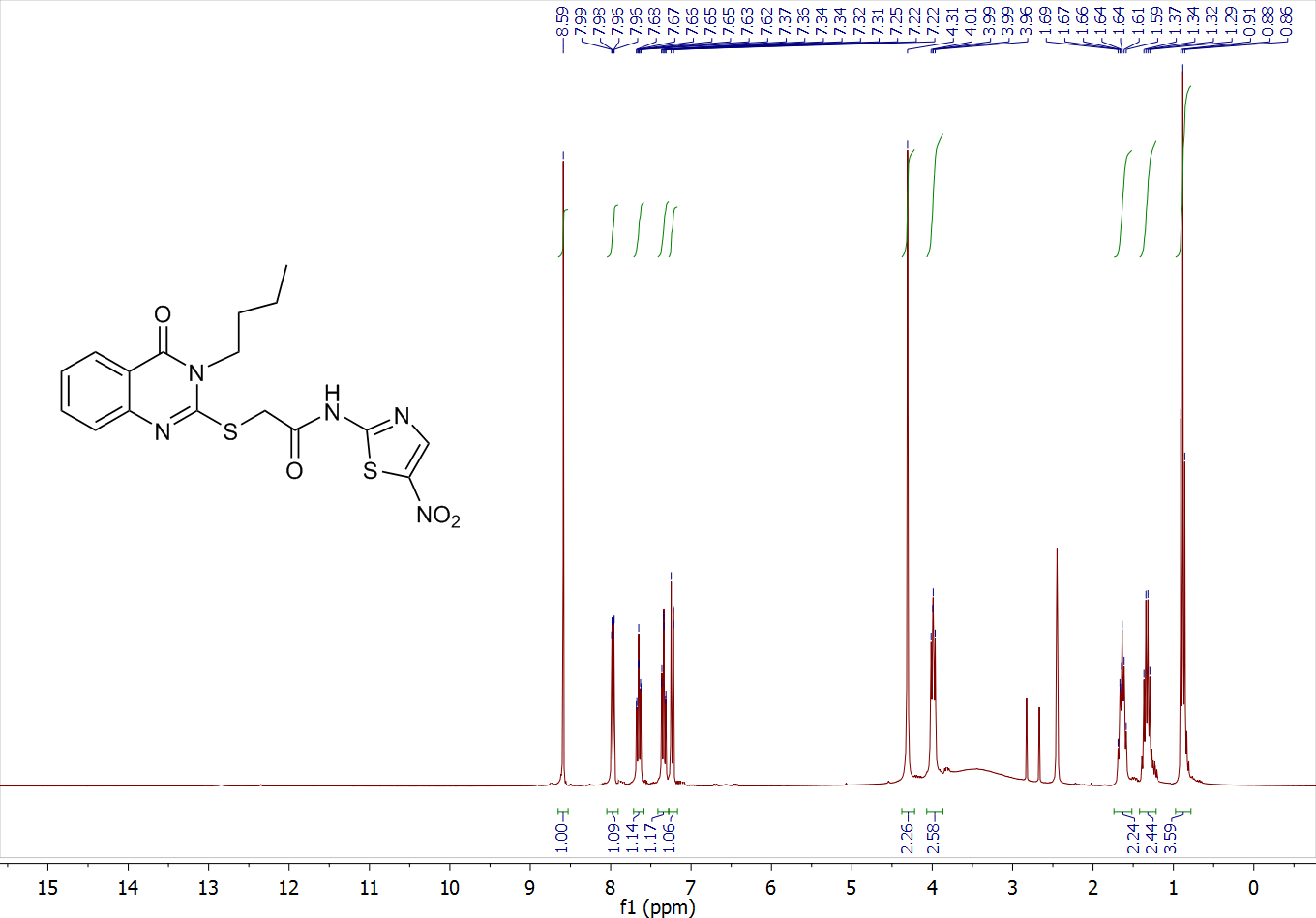


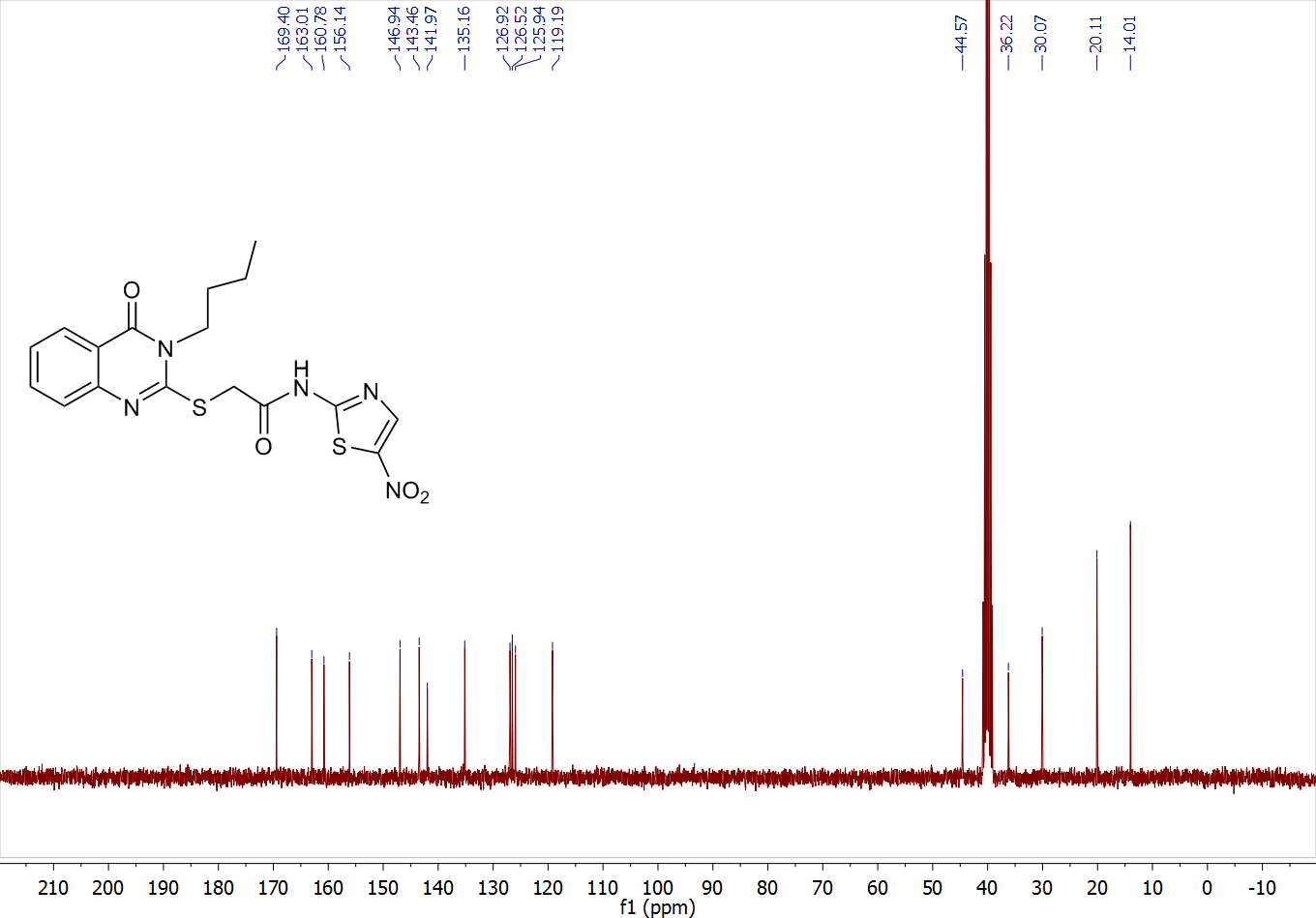


**2-((3-isobutyl-4-oxo-3,4-dihydroquinazolin-2-yl)thio)-*N*-(5-nitrothiazol-2-yl)acetamide (8l)**

**
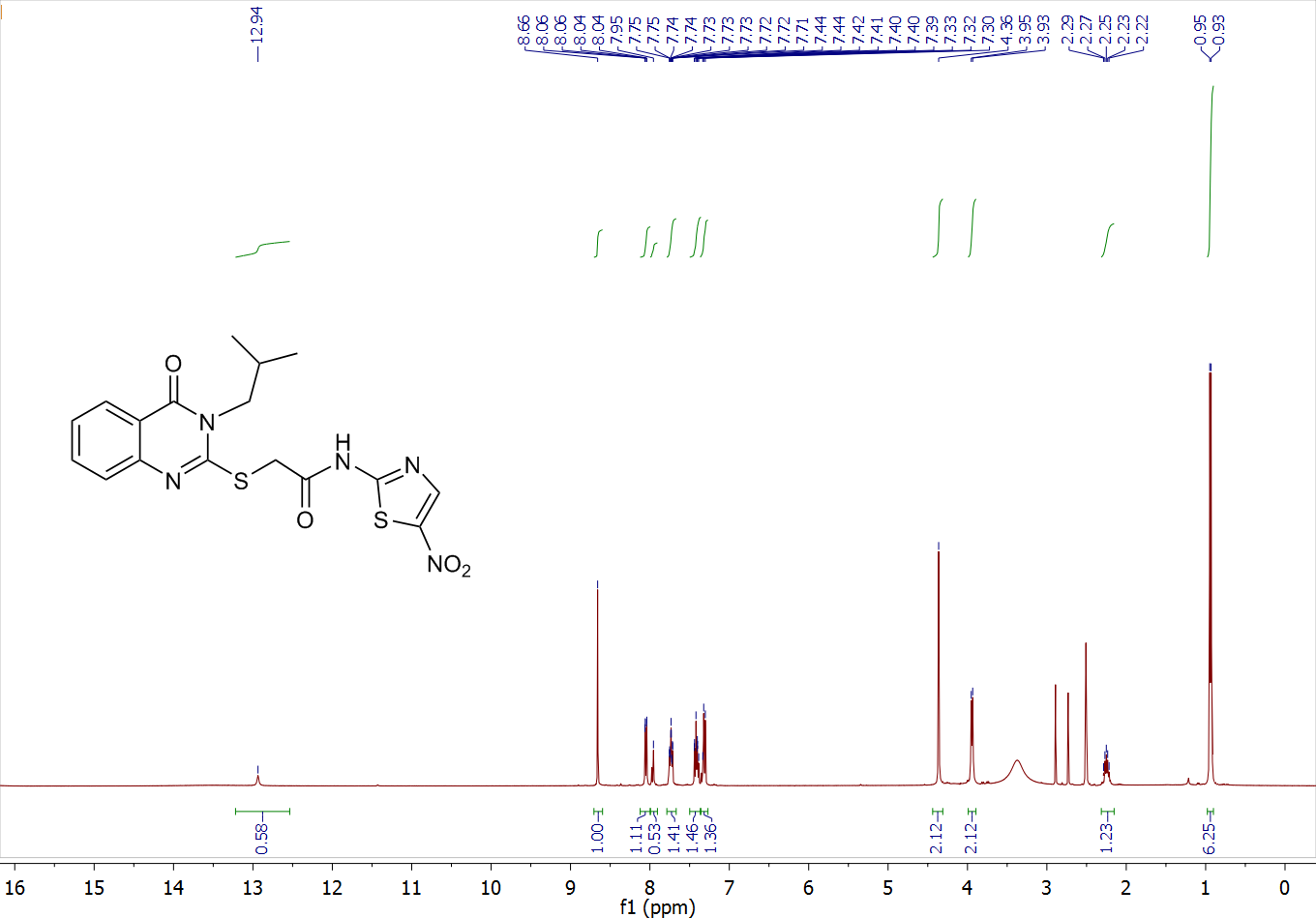
**

**
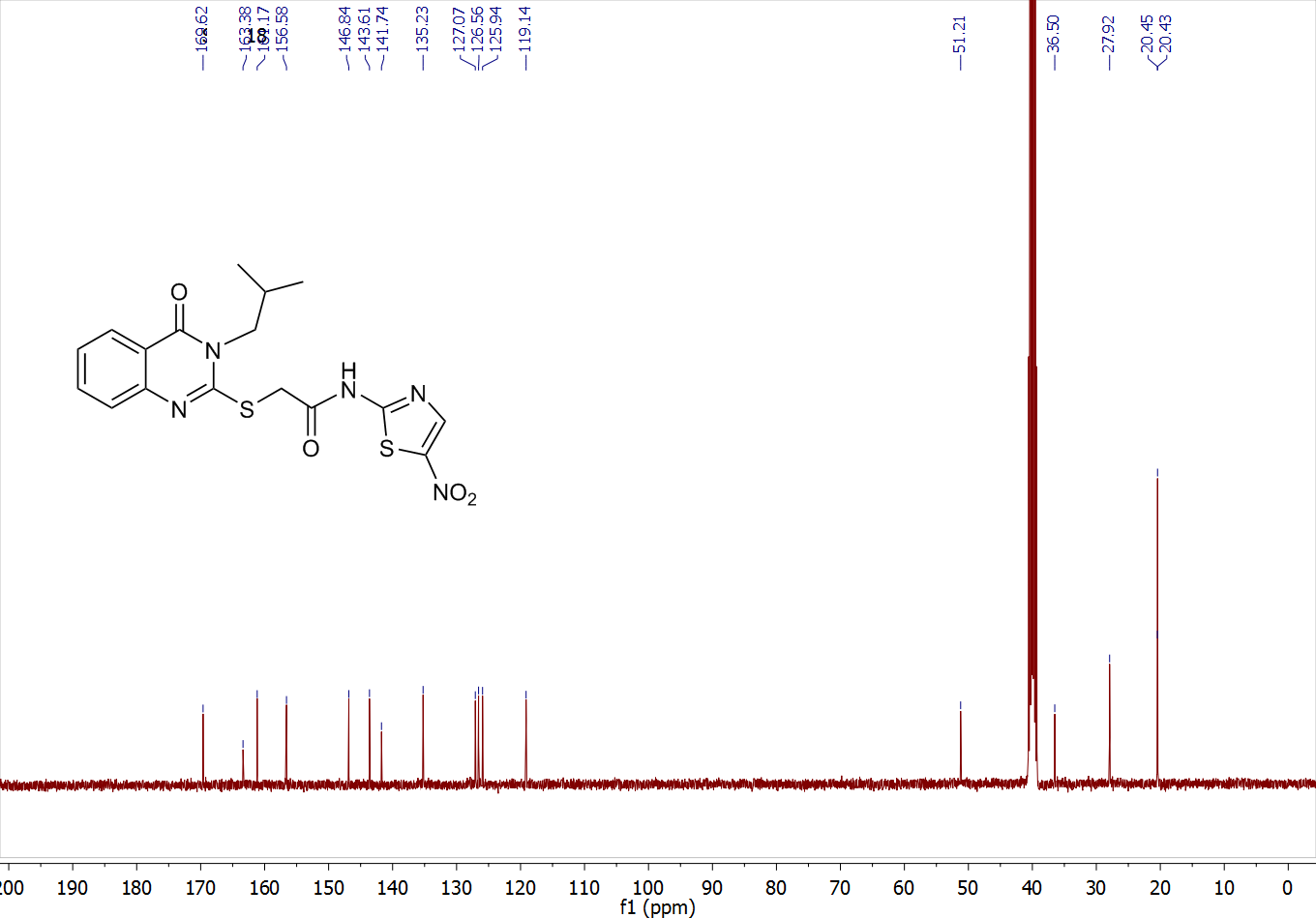
**


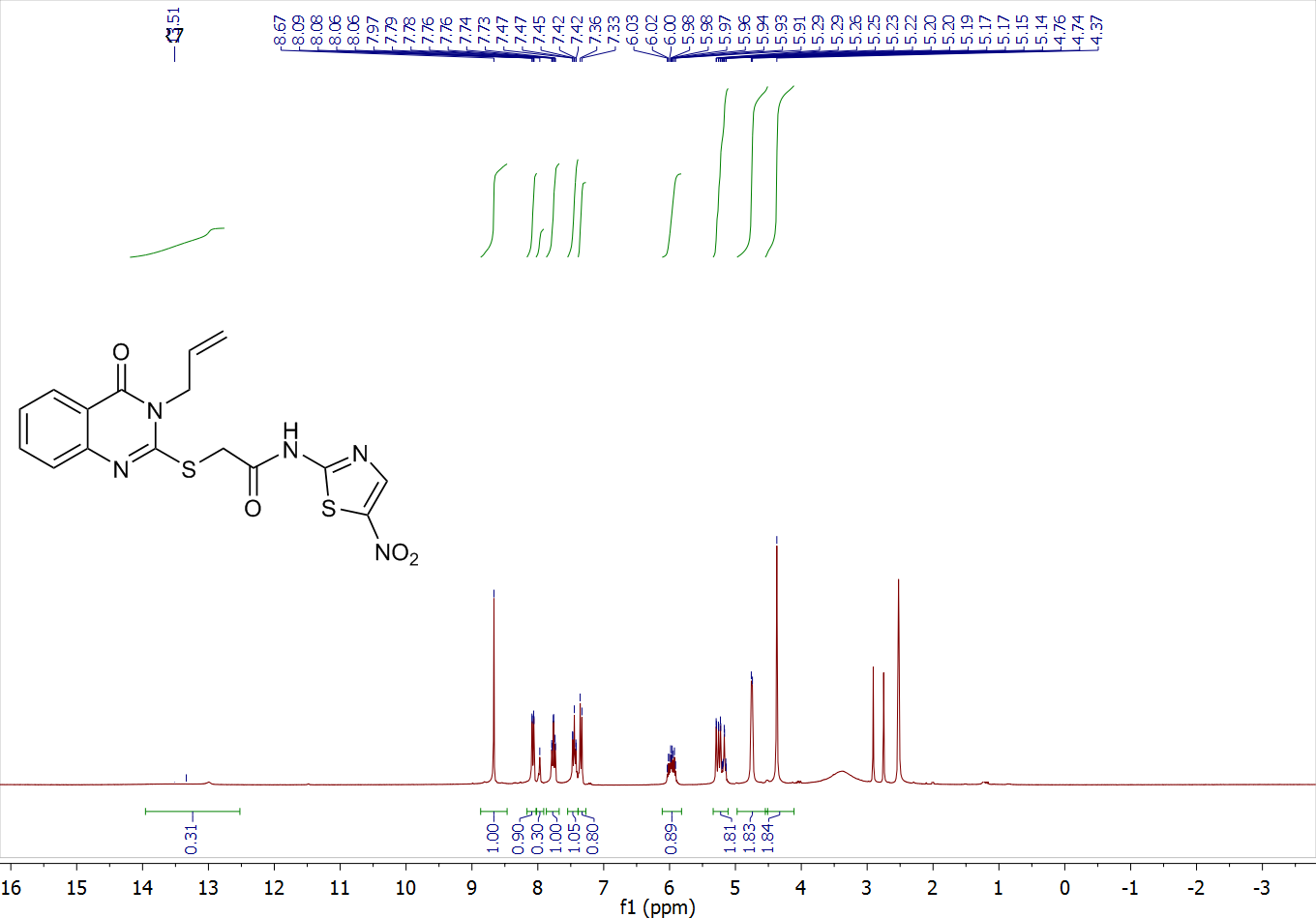
**2-((3-allyl-4-oxo-3,4-dihydroquinazolin-2-yl)thio)-*N*-(5-nitrothiazol-2-yl)acetamide (8m)**


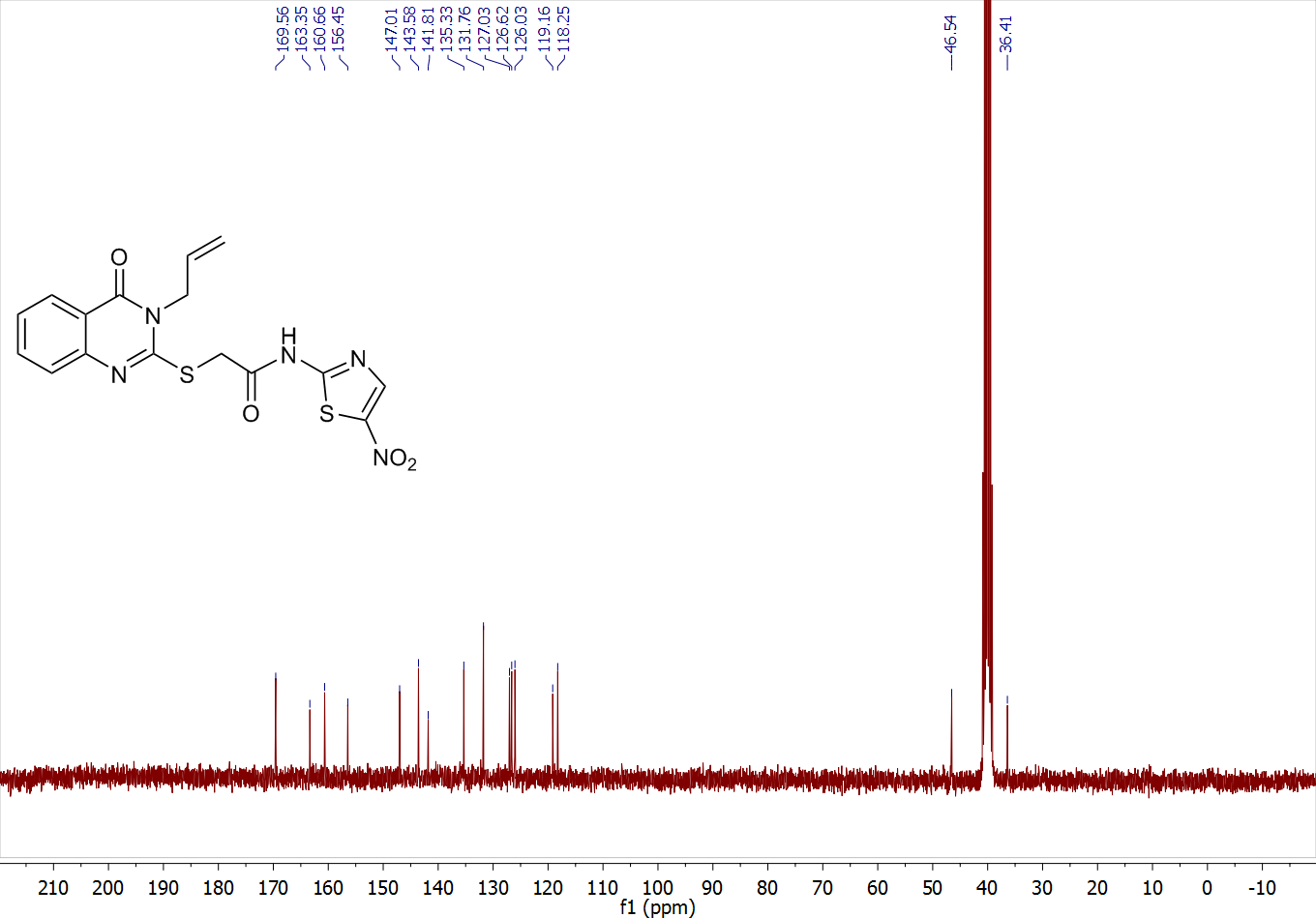


**2-((3-cyclopentyl-4-oxo-3,4-dihydroquinazolin-2-yl)thio)-*N*-(5-nitrothiazol-2-yl)acetamide (8n)**


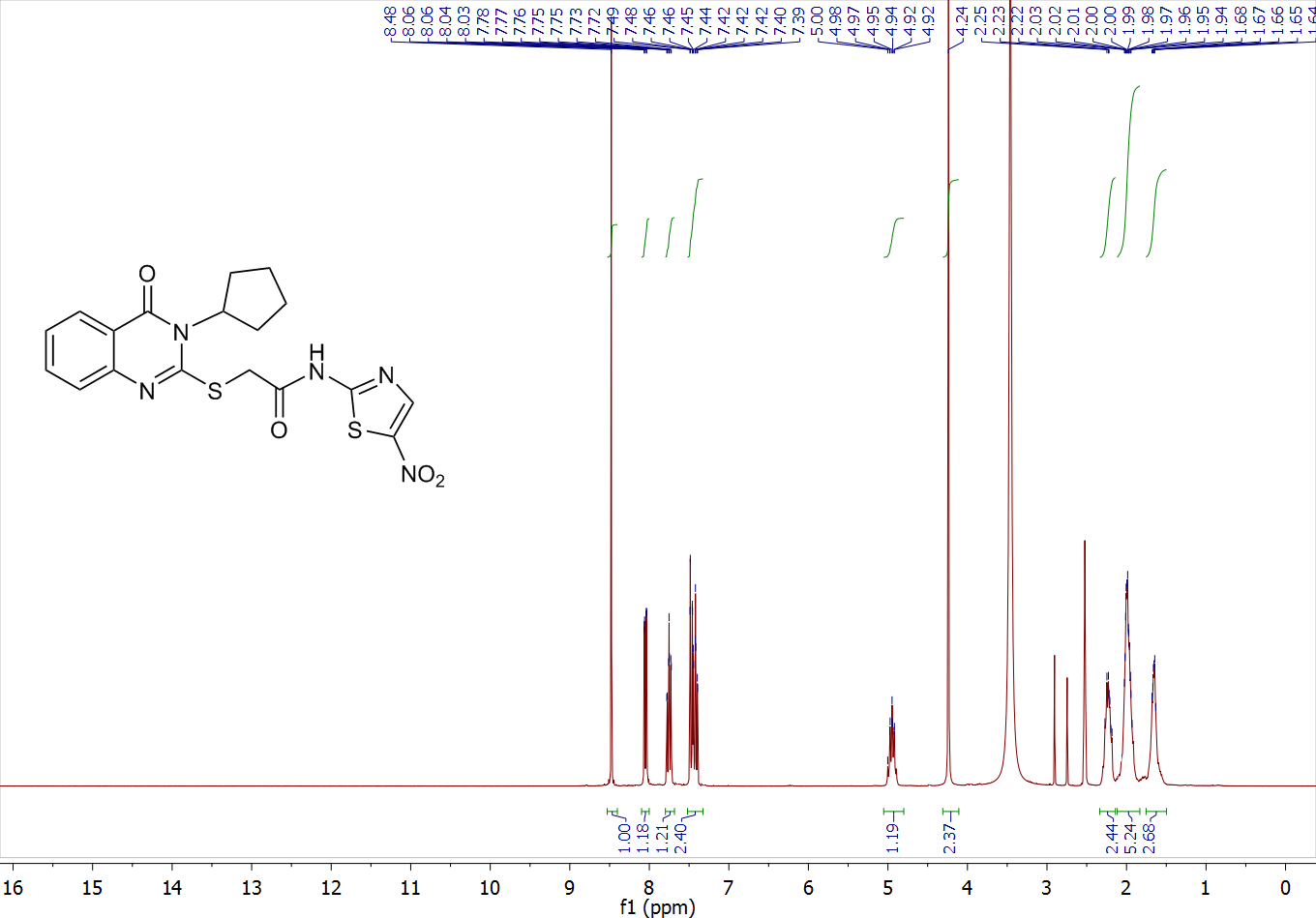


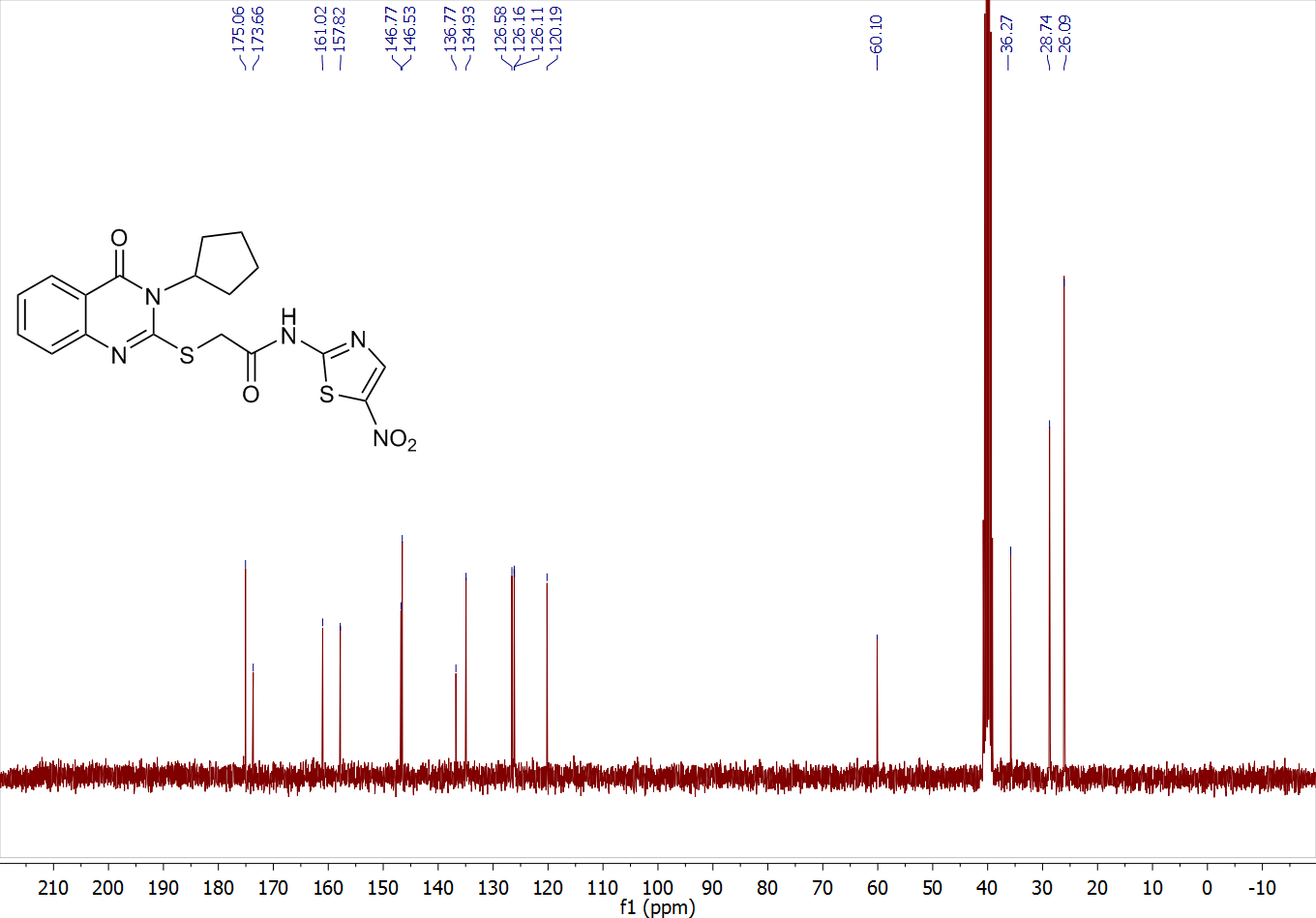

Supplement: Supplementary file 1 — Supplementary Information. [file 41598_2022_5736_MOESM1_ESM.docx]
